# Metabolic resistance to the inhibition of mitochondrial transcription revealed by CRISPR‐Cas9 screen

**DOI:** 10.15252/embr.202153054

**Published:** 2021-11-15

**Authors:** Mara Mennuni, Roberta Filograna, Andrea Felser, Nina A Bonekamp, Patrick Giavalisco, Oleksandr Lytovchenko, Nils‐Göran Larsson

**Affiliations:** ^1^ Department of Medical Biochemistry and Biophysics Karolinska Institutet Stockholm Sweden; ^2^ University Institute of Clinical Chemistry Bern University Hospital Bern Switzerland; ^3^ Mitochondrial Biology Group Max Planck Institute for Biology of Ageing Cologne Germany; ^4^ Department of Neuroanatomy Mannheim Center for Translational Neuroscience (MCTN) Medical Faculty Mannheim Heidelberg University Mannheim Germany; ^5^ Metabolomics Core Facility Max Planck Institute for Biology of Ageing Cologne Germany

**Keywords:** cancer, chemoresistance, CRISPR‐Cas9 screen, inhibitor of mitochondrial transcription, mtDNA, Cancer, Metabolism

## Abstract

Cancer cells depend on mitochondria to sustain their increased metabolic need and mitochondria therefore constitute possible targets for cancer treatment. We recently developed small‐molecule inhibitors of mitochondrial transcription (IMTs) that selectively impair mitochondrial gene expression. IMTs have potent antitumor properties *in vitro* and *in vivo*, without affecting normal tissues. Because therapy‐induced resistance is a major constraint to successful cancer therapy, we investigated mechanisms conferring resistance to IMTs. We employed a CRISPR‐Cas9 (clustered regularly interspaced short palindromic repeats)‐(CRISP‐associated protein 9) whole‐genome screen to determine pathways conferring resistance to acute IMT1 treatment. Loss of genes belonging to von Hippel–Lindau (VHL) and mammalian target of rapamycin complex 1 (mTORC1) pathways caused resistance to acute IMT1 treatment and the relevance of these pathways was confirmed by chemical modulation. We also generated cells resistant to chronic IMT treatment to understand responses to persistent mitochondrial gene expression impairment. We report that IMT1‐acquired resistance occurs through a compensatory increase of mitochondrial DNA (mtDNA) expression and cellular metabolites. We found that mitochondrial transcription factor A (TFAM) downregulation and inhibition of mitochondrial translation impaired survival of resistant cells. The identified susceptibility and resistance mechanisms to IMTs may be relevant for different types of mitochondria‐targeted therapies.

## Graphical abstract

A CRISPR‐Cas9 screen shows that mTORC1 loss increases OXPHOS and prevents IMT1‐induced cell death whereas loss of VHL confers resistance in an OXPHOS‐independent fashion.

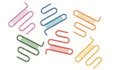

## Introduction

Mitochondria contain their own DNA (mitochondrial DNA, mtDNA), which in mammals encodes 13 subunits of the oxidative phosphorylation (OXPHOS) system, as well as mitochondrial ribosomal RNAs (rRNAs) and transfer RNAs (tRNAs) (Anderson *et al*, 1981; Bibb *et al*, 1981). Expression of mtDNA is required for proper OXPHOS function, and its disruption causes a variety of diseases, including metabolic disorders and neurodegeneration (Chinnery, 2015; Gustafsson *et al*, 2016; Kauppila *et al*, 2017). In the last decades, mitochondrial function has also been shown to be important for the development and progression of cancer (Funes *et al*, 2007; Viale *et al*, 2014; Hensley *et al*, 2016; Gammage & Frezza, 2019; Vasan *et al*, 2020). The accumulation of mtDNA mutations in cancer is a well‐established phenomenon, but it is a matter of debate whether these mutations are mere passengers or tumor drivers. The contribution of mtDNA mutations seems to be dependent on the type and stage of the tumor. The mtDNA mutation pattern in some tumors is compatible with driver properties (Gopal *et al*, 2018; Gorelick *et al*, 2021), whereas other types of mutations, for example protein‐truncating mutations, undergo negative selection in many cancers (Ju *et al*, 2014; Stewart *et al*, 2015; Yuan *et al*, 2020). In line with a proposed role for mtDNA in cancer, many groups have provided proof‐of‐concept that inhibition of OXPHOS or mitochondrial translation can indeed impair tumor growth (Škrtić *et al*, 2011; Boukalova *et al*, 2016; Reed *et al*, 2016; Molina *et al*, 2018; Lee *et al*, 2019; Shi *et al*, 2019).

As an alternative strategy to the direct inhibition of OXPHOS, we recently developed small‐molecule inhibitors of mitochondrial transcription (IMTs) that strongly impair biogenesis of the OXPHOS system (Bonekamp *et al*, 2020). The IMTs, including IMT1 used in this study, are highly specific allosteric inhibitors of the mammalian mitochondrial RNA polymerase (POLRMT) and efficiently impair the transcription of mtDNA, which, in turn, abolishes OXPHOS system biogenesis (Bonekamp *et al*, 2020). IMTs reduce the growth of human tumor cells *in vitro* and in mouse xenografts *in vivo* (Bonekamp *et al*, 2020). The importance of mtDNA transcription in tumor development is further underscored by a recent study where POLRMT levels were manipulated in human cancer cells (Zhou *et al*, 2021).

Treatment‐induced drug resistance is a major problem in cancer and it can be explained by a reprogramming of metabolism and clonal selection of therapy‐unresponsive cancer cells (Swanton, 2012). Furthermore, therapy‐resistant cancer cells, sometimes also referred to as cancer stem cells, often rely heavily on OXPHOS for their persistence (Funes *et al*, 2007; Viale *et al*, 2014; Kuntz *et al*, 2017; Valle *et al*, 2018). To gain insights into potential mechanism that can confer resistance to IMTs, we performed an unbiased whole‐genome CRISPR‐Cas9 screen (clustered regularly interspaced short palindromic repeats)‐(CRISP‐associated protein 9) of IMT1‐treated cells (Shalem *et al*, 2014; Katigbak *et al*, 2016; Sharma & Petsalaki, 2018) and also generated an IMT1‐resistant cell line through chronic IMT1 treatment. Our findings show that loss of genes in the von Hippel–Lindau (VHL) tumor suppressor and the mammalian target of rapamycin complex 1 (mTORC1) pathways confers resistance to acute IMT1 treatment, whereas further impairment of mitochondrial function, such as inhibition of mitochondrial translation and reduction of mtDNA copy number, increases sensitivity to IMT1 treatment.

## Results

### IMT1 treatment leads to progressive loss of mtDNA expression and cell death

**Figure 1:**
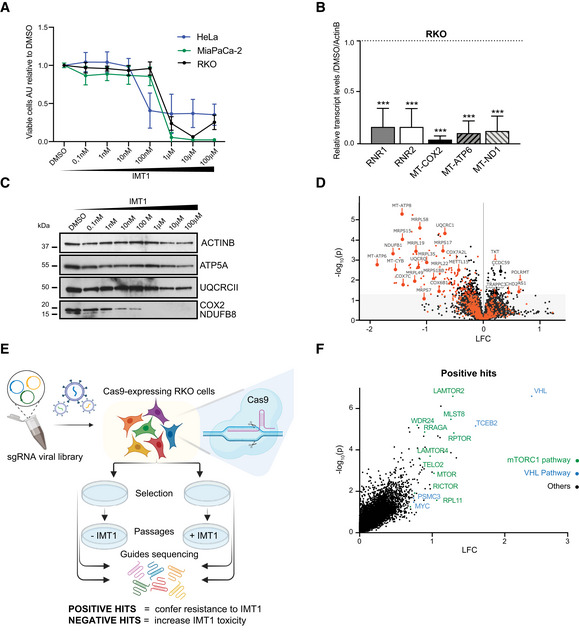
Whole‐genome CRISPR‐Cas9 (clustered regularly interspaced short palindromic repeats)‐(CRISP associated protein 9) genetic screen reveals mechanisms of resistance to IMT1 Dose–response viability curves in the presence of increasing concentrations of IMT1 for 1 week. Viable cells were counted and normalized to dimethyl sulfoxide (DMSO)‐treated controls. Data are expressed as mean ± SD of *n* = 3 independent experiments with four technical replicates. The IC_50_ values were calculated with non‐linear least squares fit, RKO: 521.8 nM, MiaPaCa‐2: 291.4 nM, and HeLa: 29.9 nM.Mitochondrial transcript levels measured by quantitative real‐time polymerase chain reaction (qRT–PCR) in RKO cells after 96 h of 1 µM IMT1 treatment. Data are relative to dimethyl sulfoxide (DMSO)‐treated controls and are expressed as mean ± SD of *n* = 4 independent experiments. Ordinary one‐way ANOVA was used for comparisons to DMSO‐treated controls (RNR1, RNR2, MT‐COX2, MT‐ATP6, and MT‐ND1: ***P < 0.0001).Representative western blot images of oxidative phosphorylation (OXPHOS) protein levels of RKO cells at increasing concentrations of IMT1. Actin B (ACTINB) is shown as loading control.Volcano plot showing proteomic changes of RKO cells treated with 1 µM IMT1 for 96 h. Data are plotted as average log_2_‐fold change (LFC) versus log_10_ of adjusted *P*‐value of dimethyl sulfoxide (DMSO)‐treated controls; mitochondrial proteins are highlighted in red.Schematic description of the experimental CRISPR‐Cas9 (clustered regularly interspaced short palindromic repeats)‐(CRISP‐associated protein 9) approach: Cas‐9 expressing‐RKO cells were transduced with a genome‐wide pooled knockout CRISPR library and passaged in the presence of either IMT1 or DMSO (dimethyl sulfoxide) for guide selection. After cell collection and DNA isolation, the guides were sequenced and quantified in each population. Created with www.BioRender.com.CRISPR (clustered regularly interspaced short palindromic repeat) guides enriched (positive hits) in IMT1‐treated RKO cells plotted as log_2_‐fold change (LFC) versus log_10_ of adjusted *P*‐value. Mammalian target of rapamycin complex 1 (mTORC1) and von Hippel–Lindau (VHL) pathway‐related genes are reported in green and blue, respectively.

**Figure EV1:**
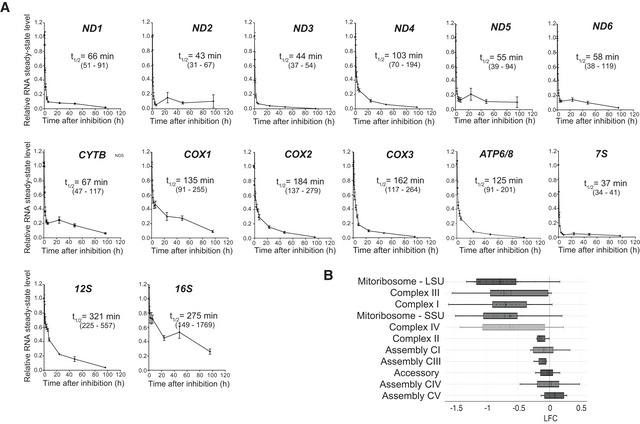
IMT1 treatment causes a fast drop in mitochondrial transcript levels Quantitative real‐time polymerase chain reaction (qRT–PCR) assessment of mitochondrial transcript levels followed over time in HeLa cells (0–96 h). Half‐life was assessed in semi‐logarithmic plot using non‐linear regression curve fit. The estimated transcript half‐lives are shown in minutes, the 95% confidence interval is indicated in brackets. Data are plotted as mean ± SEM of *n* = 4 independent experiments.Box plots showing changes in protein levels among submitochondrial compartments, according to Vögtle *et al*, 2017. Data are expressed as log_2_‐fold change (LFC) of controls (dimethyl sulfoxide (DMSO)‐treated RKO cells) from *n* = 3 independent experiments. Median (solid line) and mean (dashed line), upper and lower quartile, and 1.5× interquartile range (whiskers).

The RKO colon cancer cells are established hosts for CRISPR‐Cas9 genetic screens (Schmierer *et al*, 2017; Sayed *et al*, 2019) and we therefore characterized them further with respect to IMT1 sensitivity. We have previously demonstrated that around one third of a panel of 89 cancer cell lines are sensitive to IMT1 (Bonekamp *et al*, 2020), which reflects the heterogeneity of cancer cell lines. Besides the RKO cells, we included two additional IMT1‐sensitive cell lines, cervix (HeLa) and pancreatic cancer cell lines (MiaPaCa‐2), in this initial phase of the study. We investigated the viability of IMT1‐treated RKO, HeLa, and MiaPaCa‐2 cells and found that all had IC_50_ values below 1 µM (RKO: 521.8 nM, MiaPaCa‐2: 291.4 nM, and HeLa: 29.9 nM) (Fig 1A). There was a fast drop in mitochondrial transcript levels in IMT1‐treated HeLa cells, with half‐lives ranging between 37 and 321 min (Fig EV1A). Similarly, mitochondrial transcript levels were dramatically reduced after 96 h in IMT1‐treated RKO cells (Fig 1B). The reduction of mitochondrial gene expression resulted in a substantial decrease in the OXPHOS protein steady‐state levels, as determined by western blots of RKO cells (Fig 1C). The levels of subunits of complex I (NADH:ubiquinone oxidoreductase subunit B (NDUFB8)) and complex IV (cytochrome c oxidase subunit 2 (COX2)) dropped below the limit of detection in response to treatment with 1 µM IMT1 for 96 h, whereas subunits of complex III (ubiquinol‐cytochrome c reductase core protein 2 (UQCRCII)) and complex V (ATP Synthase F1 subunit alpha (ATP5A)) were much less affected (Fig 1C). Whole‐cell quantitative proteomic analyses showed a significant reduction of mitochondrial proteins, especially of those required for mitochondrial translation and OXPHOS, whereas proteins localized to other cellular compartments remained mostly unaffected in IMT1‐treated RKO cells (Figs 1D and EV1B and Dataset EV1). These data demonstrate that IMT1 inhibits mitochondrial gene expression in RKO cells to a similar extent as previously reported for HeLa and A2780 cells (Bonekamp *et al*, 2020).

### CRISPR‐Cas9 screening reveals that VHL and mTORC1 loss promotes resistance to IMT1

To identify factors that can modulate sensitivity to IMT1, we performed a genome‐wide CRISPR‐Cas9 screen in RKO cells. Cas9‐expressing RKO cells were transduced with a whole‐genome lentiviral single guide RNA (sgRNA) library and allowed to grow with or without 1 µM IMT1 for 10 days for clonal selection (Fig 1E). The barcoded guides were sequenced to identify genes that were enriched or depleted at the end of the experiment. Gene knockouts, which conferred resistance or increased susceptibility to IMT1 treatment, were referred to as positive and negative hits, respectively (Fig 1E). The CRISPR‐Cas9 screen revealed that VHL gene was the most significant and enriched positive hit (Fig 1F and Dataset EV2). Moreover, most other genes that increased cell survival after IMT1 treatment belonged to mTORC1 pathway (Fig 1F and Dataset EV2).

**Figure 2:**
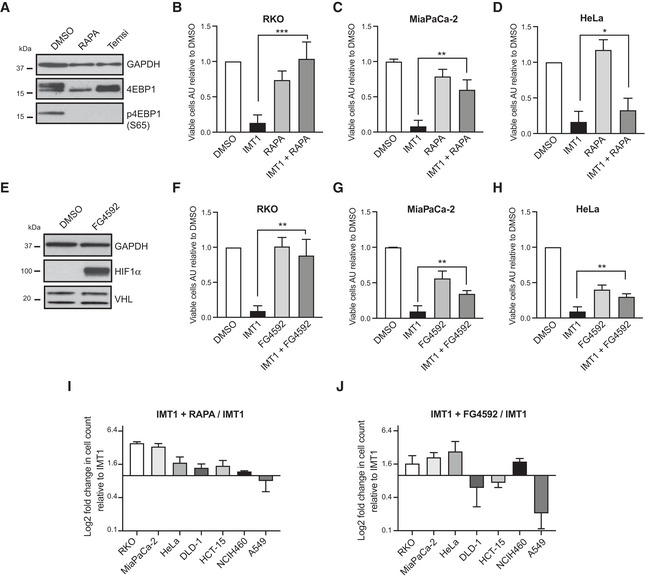
Rapamycin and FG4592 treatment rescues IMT1 toxicity ALevels of eukaryotic translation initiation factor 4E‐binding protein 1 (4EBP1) and its phosphorylation at Serine 65 in the presence of dimethyl sulfoxide (DMSO), rapamycin (100 nM), or temsirolimus (100 nM). Glyceraldehyde 3‐phosphate dehydrogenase (GAPDH) is shown as loading control.B–DViable cells count in the presence of dimethyl sulfoxide (DMSO), IMT1 alone or in combination with rapamycin in RKO (B), MiaPaCa‐2 (C), and HeLa (D) cells. Data are expressed as mean values ± SD of *n* = 6 independent experiments, each including four technical intra‐plate replicates. Statistical significance was calculated with one‐way ANOVA test. Paired IMT1 versus IMT1 + RAPA comparisons: RKO: ***P < 0.0001, MiaPaCa‐2: **P = 0.0014, and HeLa: *P = 0.0238.EWestern blot analyses showing the extent of hypoxia‐inducible factor 1α (HIF1α) stabilization and steady‐state level of von Hippel–Lindau (VHL) protein with and without the treatment with FG4592 (100 µM) in RKO cells. Glyceraldehyde 3‐phosphate dehydrogenase (GAPDH) is shown as loading control.F–HViability assessment of RKO (F), MiaPaCa‐2 (G), and HeLa (H) cells treated with dimethyl sulfoxide (DMSO), IMT1 alone and in combination with FG4592. Data are expressed as mean values ± SD of *n* = 6, 5, and 6 independent experiments for RKO, HeLa, and MiaPaCa‐2, respectively. Statistical significance was calculated with one‐way ANOVA. Paired IMT1 versus IMT1 + FG4592 comparisons: RKO: **P < 0.0028, MiaPaCa‐2: **P = 0.0011, and HeLa: *P = 0.0029.I, JLog_2_‐fold changes (LFCs) in cell viability in the presence of IMT1 and rapamycin (I) or FG4592 (J) of a panel of seven cell lines. Data are expressed as the ratio of viable cell counts in the presence of IMT1 alone and represent the mean ± SD of *n* = 6 (RKO, MiaPaCa‐2, and HeLa) and *n* = 3 (DLD‐1, HCT‐15, NCIH460, and A549) individual experiments. Graphs for each cell line are available in Fig EV2.

**Figure EV2:**
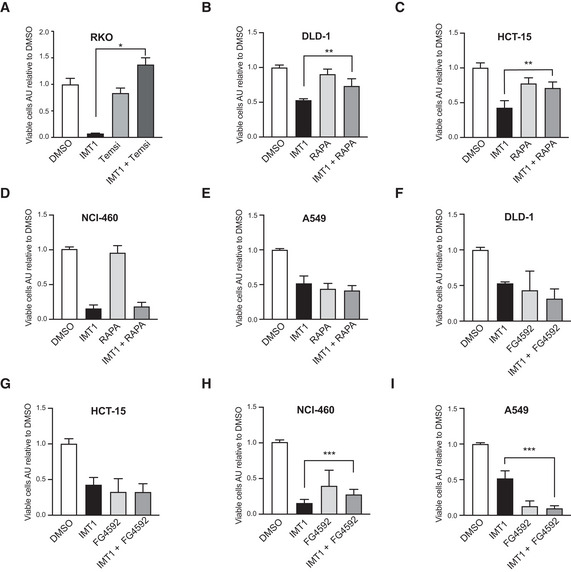
Rapamycin and FG4592 increase tolerance to IMT1 treatment in several cancer cell lines AEffect of temsirolimus alone or in combination with IMT1 on RKO cell viability. Mean ± SD of *n* = 3 independent experiments, one‐way ANOVA, IMT1 versus IMT1+ Temsirolimus: *P = 0.0278.B–ECell viability assessment of a panel of IMT1‐sensitive cancer cell lines treated with IMT1 alone and in combination with rapamycin (DLD‐1 (B), HCT‐15 (C), NCI‐460 (D), and A549 (E)). Data are expressed as mean values ± SD of *n* = 3 independent experiments, each including four technical intra‐plate replicates. Statistical significance was calculated with one‐way ANOVA test. IMT1 versus IMT1 + RAPA, DLD‐1: **P = 0.0057; HCT‐15: **P = 0.046; NCI‐460 and A549 non‐significant.F–ICell viability assessment of a panel of IMT1‐sensitive cancer cell lines treated with IMT1 alone and in combination with FG4592 (DLD‐1 (F), HCT‐15 (G), NCI‐460 (H), and A549 (I)). Data are expressed as mean values ± SD of *n* = 3 independent experiments, each including four technical intra‐plate replicates, statistical significance was calculated with one‐way ANOVA test. IMT1 versus IMT1 + FG4592, NCI‐460: ***P = 0.0003; A549: ***P < 0.0001; DLD‐1 and HCT‐15: non‐significant.

To validate the positive hits identified by the CRISPR‐Cas9 screening, we tested whether chemical perturbation of the VHL and mTORC1 pathways could increase IMT1 tolerance in RKO, MiaPaCa‐2, and HeLa cells. Treatment of RKO cells with the mTORC1 inhibitors rapamycin or temsirolimus (Sehgal *et al*, 1975; Heitman *et al*, 1991; Rini, 2008) reduced the phosphorylation of eukaryotic translation initiation factor 4E‐binding protein 1 (4EBP1), which is a downstream target of mTORC1 (Fig 2A). Both rapamycin and temsirolimus were able to rescue cell death in IMT1‐treated RKO cells (Figs 2B and EV2A). Rapamycin also increased survival of IMT1‐treated MiaPaCa‐2 (Fig 2C) and HeLa cells (Fig 2D).

Because of the well‐established role of VHL as tumor suppressor in modulating the hypoxia‐inducible factor 1α (HIF1α) (Wang & Semenza, 1993; Maxwell *et al*, 1999), we decided to use FG4592, a prolyl hydroxylase (PHD) inhibitor that stabilizes HIF1α at normal oxygen levels (Guenzler‐Pukall *et al*, 2003; Rabinowitz, 2013). Treatment of cells with FG4592 thus mimics the reduced degradation of HIF1α that occurs during hypoxia (Jain *et al*, 2016; Joharapurkar *et al*, 2018) or in the absence of the VHL protein. Treatment of RKO cells with FG4592 showed that HIF1α was stabilized under standard tissue culture conditions (Fig 2E). Notably, FG4592 treatment significantly increased the viability of RKO (Fig 2F), MiaPaCa‐2 (Fig 2G), and HeLa (Fig 2H) cells, when administered in the presence of IMT1.

To understand whether these resistance mechanisms are conserved, we included additional IMT1‐sensitive cancer cell lines that had been identified in our previous study (Bonekamp *et al*, 2020). We found that rapamycin improved tolerance to IMT1 treatment in five of the seven tested cell lines (Figs 2I and EV2B–E), whereas FG4592 partially restored viability in the presence of IMT1 of four out of seven cell lines (Figs 2J and EV2F–I). Rapamycin and FG4592 conferred resistance to IMT1 in cancer cell lines of different tissue origins, showing that these resistance mechanisms are not specific to a certain tumor type.

**Figure EV3:**
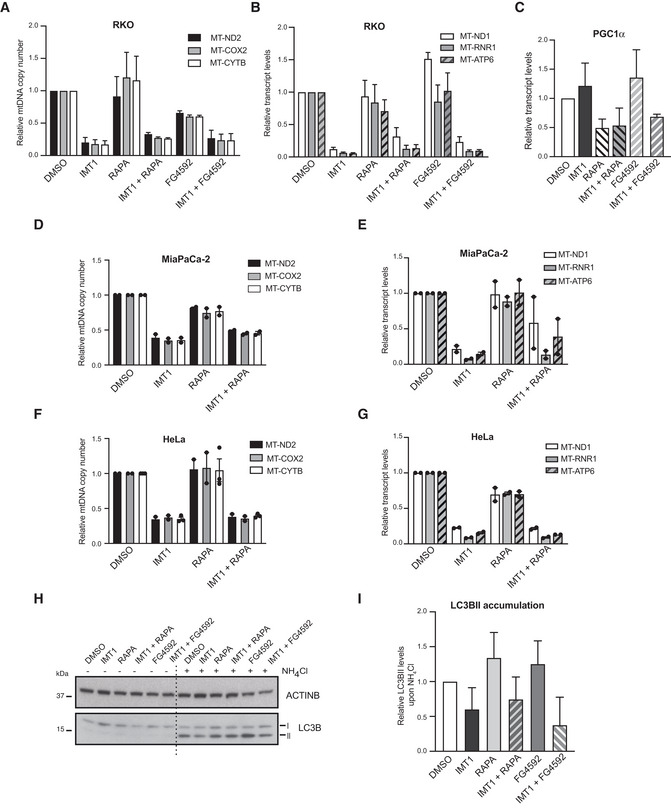
Increased mitochondrial biogenesis and changes in autophagy do not explain IMT1 resistance induced by rapamycin and FG4592 A, BQuantitative real‐time polymerase chain reaction (qRT–PCR) assessment of mitochondrial DNA (mtDNA) (A) and transcripts (B) levels in RKO cells treated with dimethyl sulfoxide (DMSO), IMT1, rapamycin, and FG4592 for 3 days. Data are relative to DMSO‐treated controls and expressed as mean ± SEM of *n* = 4 independent experiments.CRelative peroxisome proliferator‐activated receptor gamma coactivator 1 alpha (PGC1α) messenger RNA (mRNA) levels in RKO cells treated as described in A; mean ± SEM of *n* = 3 independent experiments.D–GQuantitative real‐time polymerase chain reaction (qRT–PCR) assessment of mitochondrial DNA (mtDNA) (D, F) and transcripts (E, G) levels in MiaPaCa‐2 (D, E) and HeLa (F, G) cells treated with dimethyl sulfoxide (DMSO), IMT1, rapamycin, and FG4592 for 3 days. Data are relative to DMSO‐treated controls and expressed as mean ± SEM of *n* = 2 independent experiments.HRepresentative western blot analyses of microtubule‐associated proteins 1A/1B light chain 3B (LC3BII) accumulation after blocking the autophagosome acidification with NH_4_Cl for 3 h in RKO cells in the presence of dimethyl sulfoxide (DMSO), IMT1, rapamycin, or FG4592.IDensitometric quantification of microtubule‐associated proteins 1A/1B light chain 3B (LC3BII) levels plotted as mean values ± SD from *n* = 4 independent experiments.

**Figure EV4:**
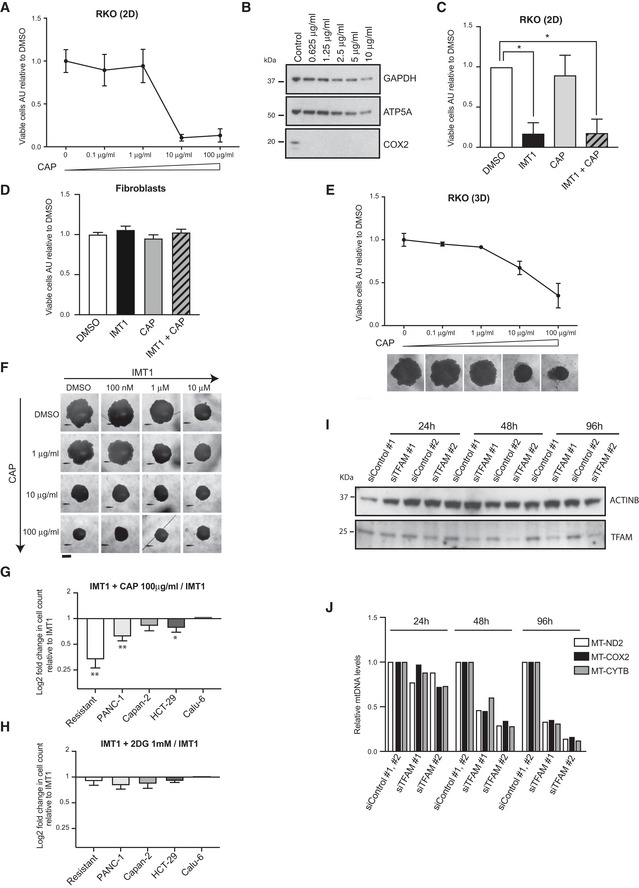
The inhibition of mitochondrial translation and decrease in mitochondrial DNA (mtDNA) copy number represent a tool to target IMT1 resistance ADose–response curves of monolayer cultures of RKO cells at increasing chloramphenicol (CAP) concentrations for 1 week, viability was determined as the ratio of dimethyl sulfoxide (DMSO)‐treated controls. Data are expressed as mean values ± SD of *n* = 3 independent experiments, each including three technical intra‐plate replicates.BRepresentative western blot assessments of mitochondrial (mt)‐encoded cytochrome c oxidase subunit 2 (COX2) protein levels at increasing chloramphenicol (CAP) concentrations, compared to ATP synthase F1 subunit alpha (ATP5A) and glyceraldehyde 3‐phosphate dehydrogenase (GAPDH) for loading reference.CViability assessment of RKO cells treated for 1 week with either dimethyl sulfoxide (DMSO), IMT1 (1 µM), chloramphenicol (CAP) (1 µg/ml) alone or in combination with IMT1. Data are expressed as mean values ± SD of *n* = 3 independent experiments, each including four technical intra‐plate replicates. Statistical significance was calculated with one‐way ANOVA test. DMSO versus IMT1: *P = 0.0159, DMSO versus IMT1+CAP: *P = 0.0264, DMSO versus CAP and IMT1 versus IMT1+CAP non‐significant.DCell viability assessment of human primary fibroblasts from two healthy individuals treated for 1 week with either dimethyl sulfoxide (DMSO), IMT1 (1 µM), chloramphenicol (CAP) (1 µg/ml) alone or in combination with IMT1. Data are the mean values ± SD of *n* = 3 independent experiments. One‐way ANOVA showed no significant difference.EDose–response curve of spheroidal growth of parental RKO cells at increasing chloramphenicol (CAP) concentrations for 2 weeks expressed as spheroid areas (mm^2^). Data represent mean values ± SD of *n* = 3 independent experiments. Representative images of the effect of serial CAP concentrations on spheroidal growth in parental RKOs are reported below the graph; scale bar: 1 mm.FDose–response spheroidal growth in RKO cells at increasing IMT1 and chloramphenicol (CAP) concentrations; scale bar: 1 mm.G, HLog_2_‐fold changes (LFCs) in viable cell counts in a panel of five IMT1‐resistant cancer cell lines in the presence of IMT1 + 100 µg/ml of chloramphenicol (CAP) (G) and IMT1 + 1 mM 2‐deoxy‐D‐glucose (2DG) (H). Data represent the mean values of *n* = 3 ±SD independent experiments; paired t‐test of IMT1 versus IMT1+CAP (100 µg/ml), resistant RKO: **P = 0.0068, PANC‐1: **P = 0.0098, Capan‐2: non‐significant and HCT‐29: *P = 0.0431, Calu‐6: non‐significant. 2DG + IMT1 versus IMT1: non‐significant.IWestern blot analyses of mitochondrial transcription factor A (TFAM) protein steady‐state levels over time after knockdown with two independent small interfering RNAs (siRNAs) against TFAM (TFAM #1 and #2) or controls (Control #1, #2) in IMT1‐resistant RKO cells.JChanges in mitochondrial DNA (mtDNA) levels after mitochondrial transcription factor A (TFAM) downregulation performed as in panel I.

### Rapamycin and FG4592 confer resistance to IMT1 through different mechanisms

We next assessed the effect of rapamycin and FG4592 on mitochondrial function. We first tested whether the two compounds had an impact on mitochondrial biogenesis, but found no significant increase in the levels of mtDNA (Fig EV3A) or mitochondrial transcripts (Fig EV3B) in RKO cells treated with IMT1 in combination with rapamycin or FG4592. In line with this, there was no induction of the peroxisome proliferator‐activated receptor gamma coactivator 1 alpha (PGC1α) expression in either of these conditions (Fig EV3C). Similarly, rapamycin treatment had no effect on mtDNA (Fig EV3D and F) and mitochondrial transcript levels (Fig EV3E and G) in IMT1‐treated MiaPaCa‐2 and HeLa cells.

**Figure 3:**
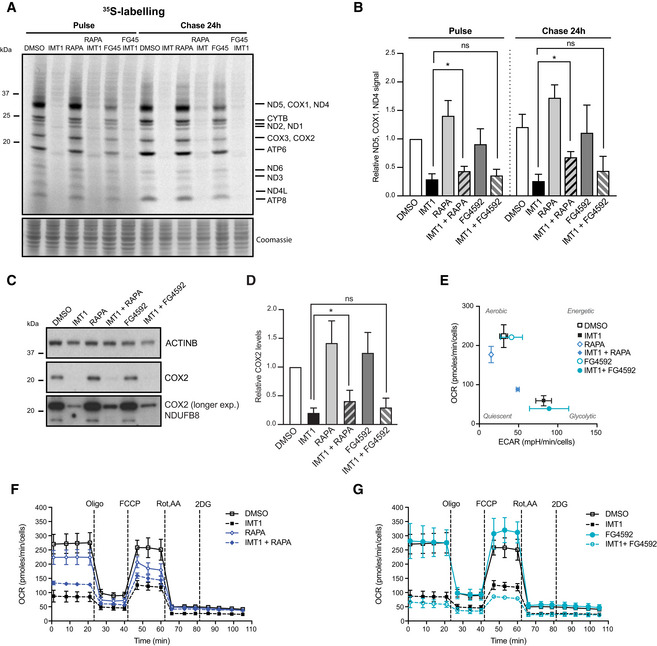
Rapamycin, but not FG4592, treatment sustains oxidative phosphorylation (OXPHOS) function in the presence of IMT1 A
^35^S‐labeling of newly synthesized mitochondrial‐encoded proteins of RKO cells treated either with dimethyl sulfoxide (DMSO), IMT1, rapamycin, and FG4592 alone or in combination with IMT1. Left: pulse labeling; right: 24‐h chase in normal medium. Putative mitochondrial proteins are reported on the right‐hand side of the gel, molecular weights on the left. Coomassie staining shows equal protein loading; the image is representative of *n* = 4 independent experiments.BDensitometric quantification of the ND5, COX1, ND4 band from *n* = 4 ^35^S‐labeling experiments. Data are expressed as mean values ± SD. Statistical significance was calculated with one‐way ANOVA test. Paired comparisons: IMT1 versus IMT1 + RAPA pulse: *P = 0.015; IMT1 versus IMT1 + RAPA chase: *P = 0.046; IMT1 versus IMT1 + FG4592 pulse and chase: non‐significant.CWestern blot analyses of oxidative phosphorylation (OXPHOS) protein steady‐state levels of RKO cells after treatment with dimethyl sulfoxide (DMSO), IMT1, rapamycin, rapamycin with IMT1, FG4592, and FG4592 with IMT1. Actin B (ACTINB) is shown as loading control.DDensitometric quantification of cytochrome c oxidase subunit 2 (COX2) protein levels from *n* = 4 experiments. Data are expressed as mean values ± SD. Statistical significance was calculated with one‐way ANOVA. IMT1 versus IMT1 + RAPA: *P = 0.016; IMT1 versus IMT1 + FG4592: non‐significant.EBioenergetic profiles measured with Seahorse flux analyzer showing the contribution of oxygen consumption rate (OCR) and extracellular acidification rate (ECAR) to cellular bioenergetics upon the aforementioned treatments. Data represent the mean ± SEM of *n* = 3 independent experiments.F, GCellular oxygen consumption rate (OCR) measured with Seahorse extracellular flux analyzer after sequential addition of different modulators of mitochondrial function. RKO cells were treated with dimethyl sulfoxide (DMSO), rapamycin (F), or FG4592 (G) in the presence or absence of IMT1. Data are expressed as the mean ± SEM of *n* = 3 independent experiments with six technical replicates. Statistical significance was calculated with one‐way ANOVA test. DMSO‐treated controls versus: IMT1: *P* = 0.0008; RAPA: *P* = 0.1578; RAPA + IMT1: *P* = 0.0034; FG4592: *P* = 0.872; FG4592 + IMT1: *P* = 0.0029. Paired t‐test of IMT1 versus RAPA + IMT1: *P* = 0.034; IMT1 versus FG4592 + IMT1: non‐significant.

Next, we assessed the production rate and the stability of mtDNA‐encoded OXPHOS proteins by performing ^35^S‐labeling of newly synthesized mitochondrial proteins in whole cells. We found a mild, but significant, increase in both the production rate (pulse) and the stability (chase) of newly synthesized mitochondrial proteins after IMT1 treatment in the presence of rapamycin (Fig 3A and B). In contrast, treatment of RKO cells with IMT1 in the presence of FG4592 did not result in significant changes in protein production rates or stability (Fig 3A and B). Consistent with this, the steady‐state levels of the mitochondrial‐encoded COX2 protein (Fig 3C and D) and oxygen consumption rates (OCRs) were higher if the IMT1 treatment was combined with rapamycin (Fig 3E and F), whereas the combination with FG4592 had no such effect (Fig 3C, D, E and G). The bioenergetic profile graph, which reports the contribution of OCR and extracellular acidification rate (ECAR) to cellular bioenergetics, showed a shift toward glycolysis in the presence of IMT1 (Fig 3E). However, the shift toward glycolysis was smaller, if IMT1 was combined with rapamycin, in comparison with IMT1 alone (Fig 3E). Based on these data, we conclude that rapamycin and FG4592 confer resistance to IMT1 treatment through distinct mechanisms.

Given the relationship between cellular energy homeostasis, nutrient availability, and autophagy (Yang *et al*, 2019), we determined whether changes in autophagy could account for the increased tolerance to IMT1 in the presence of rapamycin and FG4592. We estimated the cellular autophagic flux by assessing the accumulation of the microtubule‐associated proteins 1A/1B light chain 3B (LC3BII) after the inhibition of lysosomal acidification with ammonium chloride and observed a marked decrease in LC3BII levels in the presence of IMT1 (Fig EV3H and I). We found that neither rapamycin nor FG4592 could normalize the reduced autophagic flux caused by IMT1 treatment. We therefore conclude that the protective action of rapamycin and FG4592 is likely not caused by the normalization of autophagy in IMT1‐treated cells (Fig EV3H and I).

### Dose‐escalated chronic IMT1 treatment confers drug resistance to RKO cells

**Figure 4:**
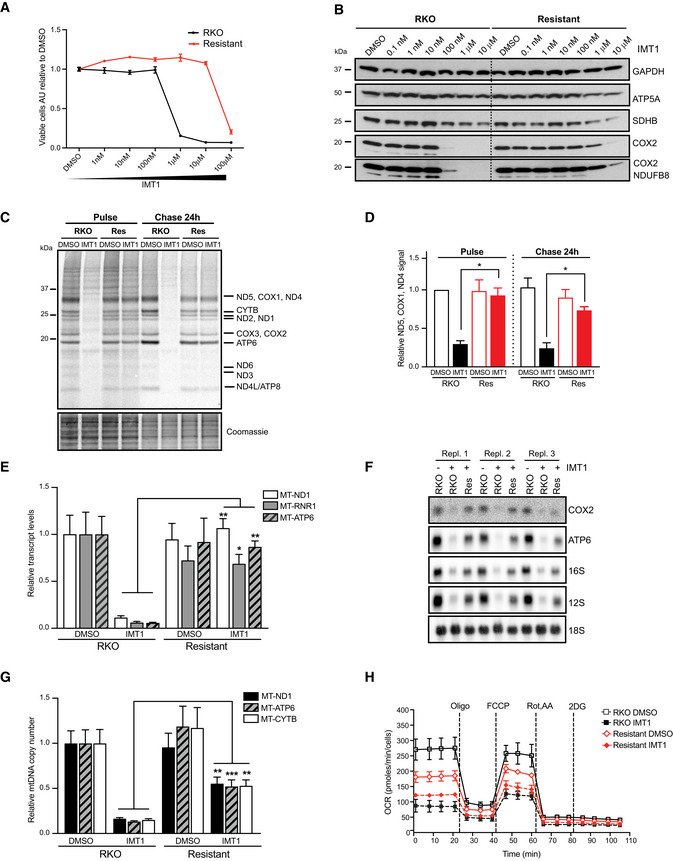
Chronic dose‐escalated IMT1 treatment confers resistance to RKO cells through an upregulation of mitochondrial function Differences in sensitivity to increasing IMT1 doses between RKO cells and resistant RKO cells. The graph shows mean values ± SEM of *n* = 3 independent experiments.Western blot analyses of oxidative phosphorylation (OXPHOS) protein steady‐state levels at increasing concentrations of IMT1 of RKO (left) and IMT1‐resistant RKO cells (right). The samples were collected and analyzed after 8 weeks of chronic IMT1 treatment of the resistant line; glyceraldehyde 3‐phosphate dehydrogenase (GAPDH) is shown as loading control.
^35^S‐labeling of newly synthesized mitochondrial‐encoded proteins in RKO and IMT1‐resistant RKO cells treated either with dimethyl sulfoxide (DMSO) or with 1 µM IMT1 for 4 days. Left: Pulse labeling, right: 24‐h chase in normal medium. Putative mitochondrial proteins are reported on the right‐hand side of the gel, molecular weights on the left. Coomassie staining shows protein loading.Densitometric quantification ± SD of ND5, COX1, ND4 band from *n* = 4 independent experiments. Statistical significance was calculated with paired t‐test: RKO + IMT1 versus resistant + IMT1, pulse: *P = 0.0139, chase: *P = 0.0368.Quantitative real‐time–polymerase chain reaction (qRT–PCR) relative quantification of mitochondrial transcript levels of RKO and IMT1‐resistant RKO cells treated with dimethyl sulfoxide (DMSO) or IMT1 for 96 h. Data represent the mean values ± SEM of *n* = 5 experiments. Statistical significance was calculated with one‐way ANOVA test. RKO + IMT1 versus Resistant + IMT1: MT‐ND1 **P = 0.0092, MT‐RNR1 *P = 0.0112, MT‐ATP6 **P = 0.0022.Northern blot analyses of mitochondrial transcripts of RKO cells treated with dimethyl sulfoxide (DMSO) (RKO, “IMT1 –”), IMT1 (RKO “+ IMT1”), and IMT1‐resistant cells (Res, “IMT1 +”). *n* = 3 independent experiments (Repl. 1, 21, and 3) were probed for COX2, ATP6, 16S and 12S mtRNA; the nuclear‐encoded 18S RNA is shown as loading control.Relative mitochondrial DNA (mtDNA) copy number measured in RKO and IMT1‐resistant cells treated with IMT1 for 96 h. Data are expressed as average from *n* = 3 experiments ± SEM. Statistical significance was calculated with one‐way ANOVA test. RKO + IMT1 versus Resistant + IMT1: MT‐ND1 **P = 0.0035, MT‐ATP6 ***P = 0.0002, MT‐CYTB **P = 0.0028.Cellular oxygen consumption rate (OCR) measured by Seahorse extracellular flux analysis after sequential addition of different modulators of mitochondrial function in RKO and IMT1‐resistant RKO cells treated with dimethyl sulfoxide (DMSO) or IMT1. Data are expressed as the mean ± SEM of *n* = 3 independent experiments with six technical replicates. Statistical significance was calculated with one‐way ANOVA test. RKO DMSO versus RKO + IMT1: *P* = 0.0008; RKO DMSO versus resistant DMSO: *P* = 0.0343; RKO DMSO versus resistant + IMT1: *P* = 0.0092. Paired t‐test of RKO + IMT1 versus resistant + IMT1: *P* = 0.135.

The CRISPR‐Cas9 screen identified cellular responses promoting survival under acute inhibition of mitochondrial transcription. In order to understand the long‐term cellular adaptations to the inhibition of mitochondrial transcription, which often occurs in disease states, we exploited an escalated‐dose treatment approach (McDermott *et al*, 2014). We exposed RKO cells to sublethal IMT1 doses and gradually increased the drug concentration over several weeks to generate resistant lines. The IMT1 effect on viability and OXPHOS protein abundance was assessed at different time points during the generation of the resistant lines. When compared to the original RKO cell line, the cells chronically exposed to IMT1 for 2 months showed increased tolerance to IMT1. Exposure to 1 µM IMT1 for 7 days was lethal for RKO cells, but was not toxic for the resistant line (Fig 4A). In comparison with the original RKO cells, the resistant cells maintained OXPHOS protein levels (COX2 and NDUFB8) at high IMT1 doses better (Fig 4B). While IMT1 completely abolished mitochondrial translation in RKO cells (Fig 4C and D, pulse), the resistant cells maintained mitochondrial translation rates at higher levels in the presence of IMT1, as shown in the ^35^S‐labeling experiment (Fig 4C and D, pulse). No differences in protein stability were observed between the dimethyl sulfoxide (DMSO)‐treated resistant and parental RKO cells in the 24‐h chase experiment (Fig 4C and D, chase). The mtDNA transcript levels in IMT1‐treated RKO cells were drastically reduced, whereas IMT1‐treated resistant cells had near‐normal levels of transcripts in comparison with untreated RKO cells (Fig 4E and F). Interestingly, the mtDNA levels were also higher in the IMT1‐treated resistant line compared with IMT1‐treated parental RKO cells, and reached levels corresponding to ~50% of the mtDNA levels of the controls (Fig 4G). The characterization of cellular bioenergetics performed with the Seahorse flux analyzer revealed that DMSO‐treated resistant cells had lower basal OCR than DMSO‐treated parental RKO cells (Fig 4H). While IMT1 treatment resulted in a strong reduction of respiration rates in RKO cells, the drop in OCR was smaller in IMT1‐treated resistant cells (Fig 4H). These data show that the resistant cells can maintain higher mtDNA levels than the original RKO cells when treated with IMT1, which allow them to maintain near‐normal levels of mtDNA‐encoded transcripts and mitochondrial proteins, resulting in a partial rescue of OCR.

### Increased glycolysis, reduced drug uptake, or mutations in the drug target do not explain IMT1‐induced resistance

**Figure 5:**
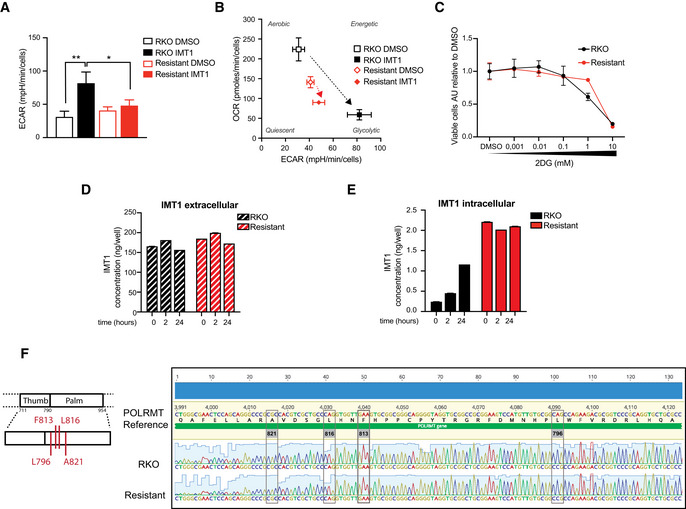
Increased glycolysis, reduced drug uptake, or mutations in the drug target do not explain IMT1‐induced resistance AExtracellular acidification rate (ECAR) measured by Seahorse extracellular flux analysis in dimethyl sulfoxide (DMSO) and IMT1‐treated RKO and IMT1‐resistant cells. Data are expressed as the mean ± SEM of *n* = 3 independent experiments with six technical replicates. Statistical significance was calculated with one‐way ANOVA test for multiple comparisons. RKO DMSO versus RKO + IMT1: **P = 0.0019; RKO + IMT1 versus resistant + IMT: *P = 0.0214; RKO DMSO versus resistant DMSO: non‐significant.BBioenergetic profiles measured with Seahorse flux analyzer, showing the contribution of oxygen consumption rate (OCR) and extracellular acidification rate (ECAR) to cellular bioenergetics in RKO and IMT1‐resistant RKO cells treated with and without IMT1. Mean of *n* = 3 independent experiments ± SEM.CDose–response viability curves during treatment with serial dilution of IMT1 for 1 week in RKO and IMT1‐resistant RKO cells. Data are expressed as the mean ± SD of *n* = 3 experiments.D, EIMT1 extracellular (D) and extracellular (E) concentrations measured in the medium at 0, 2, and 24 h of IMT1 treatment in RKO and IMT1‐resistant RKO cells. Mean values ± SEM of *n* = 3 experiments.FSchematic representation of the IMT1‐binding region on the human mitochondrial RNA polymerase (POLRMT) sequence, adapted from (Hillen *et al*, 2018). Previously identified mutations conferring resistance to IMT1 are reported in red (Bonekamp *et al*, 2020). Representative results of *n* = 3 independent sequencing experiments of the POLRMT gene in RKO and IMT1‐resistant RKO cells at the region of interest.

Reliance on glycolysis is one of the mechanisms by which cancer cells can compensate for impaired OXPHOS. To understand whether increased glycolysis plays a role in IMT1‐induced resistance, we measured ECAR, but found no significant differences between the RKO and IMT1‐resistant RKO cells (Fig 5A). IMT1 treatment caused a twofold increase in ECAR in the RKO cells, whereas no difference was found in the IMT1‐resistant RKO cells (Fig 5A). The bioenergetic profile graph revealed a mild switch toward glycolysis in the IMT1‐treated resistant cells (Fig 5B). In agreement with these data, we found no difference in the sensitivity to treatment with 2‐deoxy‐D‐glucose (2DG), a well‐known glycolysis inhibitor (Woodward & Hudson, 1954), between RKO and IMT1‐resistant RKO cells (Fig 5C). Therefore, these findings exclude increased reliance on glycolysis as an important mechanism for IMT1‐acquired resistance.

Drug resistance is often caused by multiple drug resistance (MDR) mechanisms (Szakács *et al*, 2006). Common MDR mechanisms include reduced uptake and increased efflux of drugs from the cell through the plasma membrane by ATP‐binding cassette (ABC) transporters (Szakács *et al*, 2006; Swanton, 2012). To investigate this possibility further, we measured the levels of IMT1 in RKO and IMT1‐resistant RKO cells, and found no differences in the extracellular concentration (Fig 5D), whereas the intracellular concentration was higher in the resistant line (Fig 5E), likely because these cells were continuously maintained by culture in IMT1‐containing medium. These findings exclude decreased cellular absorption or increased excretion of IMT1 as a resistance mechanism.

We have previously performed cryo‐electron microscopy (cryo‐EM) studies and identified a specific pocket of POLRMT where the IMTs bind (Bonekamp *et al*, 2020). An unbiased mutagenesis screen has shown that substitutions of four amino acids (L796, F813, L816, and A821) clustered in this pocket confer resistance to IMTs (Bonekamp *et al*, 2020). We therefore sequenced this *POLRMT* region in the IMT1‐resistant RKO cells, but found no mutations (Fig 5F). These findings support the conclusion that the dose‐escalation protocol we used to generate IMT1‐resistant RKO cells did not promote selection of clones of cells with mutations in *POLRMT* that compromise IMT1 binding.

### The resistant cell line maintains higher metabolite levels in the presence of IMT1

**Figure 6:**
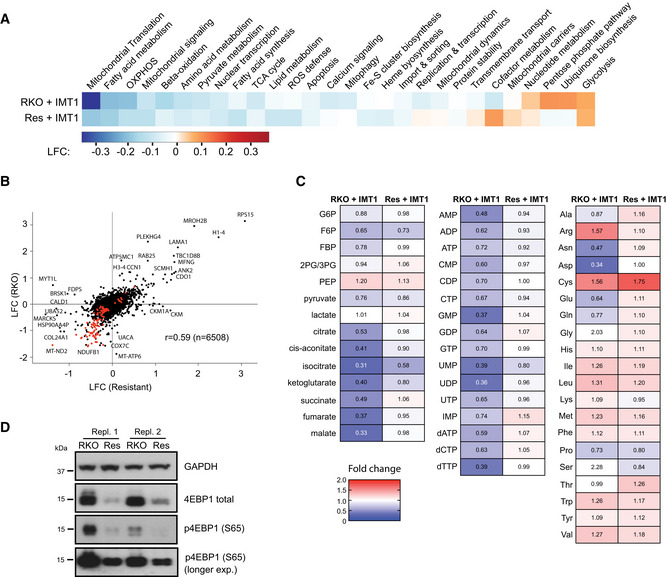
Cellular metabolite levels are maintained in IMT1‐induced resistant cells Metabolic Atlas pathway analysis of proteomic changes of RKO and IMT1‐resistant RKO cells treated with 1 µM IMT1 for 96 h. Data are expressed as average log_2_‐fold change (LFC) of controls (dimethyl sulfoxide (DMSO)‐treated RKO cells).Comparison between proteomic analyses of parental RKO and resistant cells treated with 1 µM IMT1 for 96 h (r: sample correlation coefficient). Data are expressed as average log_2_‐fold change (LFC) of controls (DMSO‐treated RKO cells). Mitochondrial proteins are reported in red.Fold changes in nucleotide, central carbon metabolite, and amino acid levels after IMT1 treatment in RKO and IMT1‐resistant RKO cells. Metabolite levels are expressed as fold of dimethyl sulfoxide (DMSO)‐treated RKO cells (mean IMT1‐treated/control, *n* = 3 experiments). Dark blue, minimum (0), dark red, maximum (2). G6P, glucose‐6‐phosphate; F6P, fructose‐6‐phosphate; FBP, fructose‐1,6‐bisphosphate; 3PG, 3‐phosphoglycerate; PEP, phosphoenolpyruvate.Western blot analyses of total eukaryotic translation initiation factor 4E‐binding protein 1 (4EBP1) and its phosphorylated form levels of RKO and IMT1‐resistant RKO cells, two representative experiments are shown (Repl. 1 and 2). Glyceraldehyde 3‐phosphate dehydrogenase (GAPDH) is shown as loading control.

Having excluded mutations of the POLRMT target enzyme and MDR mechanisms as explanations for IMT1 resistance, we focused on identifying metabolic adaptations to IMT1 chronic treatment. First, we performed whole‐cell proteomic analyses in RKO cells and IMT1‐resistant RKO cells treated with 1 µM IMT1 for 96 h and found quite similar proteomic changes (Pearson’s *r* = 0.59; Fig 6A and B and Dataset EV1), despite the previously observed differences in cell survival (Fig 4A). The proteins with changed expression (Fig 6B and Dataset EV1) and the involved metabolic pathways (Fig 6A) were similar in RKO cells and IMT1‐resistant RKO cells, although the changes were less pronounced in the resistant cells. As reported in our previous paper, IMT1 induces a cellular energy crisis leading to depletion of metabolites and eventually cell death (Bonekamp *et al*, 2020). We therefore performed metabolic analyses and found a strong reduction in cellular nucleotide levels, tricarboxylic acid (TCA) cycle intermediates and, to a lesser extent, amino acids in RKO cells treated with IMT1 in comparison with DMSO‐treated controls (Fig 6C and Dataset EV3). However, metabolites were maintained at higher levels after IMT1 treatment of IMT1‐resistant RKO cells in comparison with IMT1 treatment of RKO cells (Fig 6C and Dataset EV3), thus preventing a critical decline of cellular metabolism.

Because mTORC1 inhibition and HIF1α stabilization confer resistance to IMT1‐sensitive cells (Figs 2B–D and EV2A–C), we investigated whether these pathways were affected in the IMT1‐resistant RKO line. HIF1α was not detected in the proteomic analysis of resistant cells (Dataset EV1), suggesting that HIF1α stabilization does not explain resistance. No significant changes in mTORC1‐related proteins were identified in the same dataset (Dataset EV1). However, changes in mTORC1 activity may occur without the protein levels being affected and we therefore assessed the extent of 4EBP1 phosphorylation as a readout of mTORC1 activity. Interestingly, IMT1‐resistant RKO cells showed reduced phosphorylation of the mTORC1 target 4EBP1 in comparison with RKO cells (Fig 6D). Rapamycin and temsirolimus promoted survival of cells acutely exposed to IMT1 (Figs 2B–D and EV2A–C) and our results argue that mTORC1 inhibition may also be of importance in chronic IMT1 resistance.

### Inhibition of mitochondrial translation and TFAM downregulation can overcome IMT1 resistance

**Figure 7:**
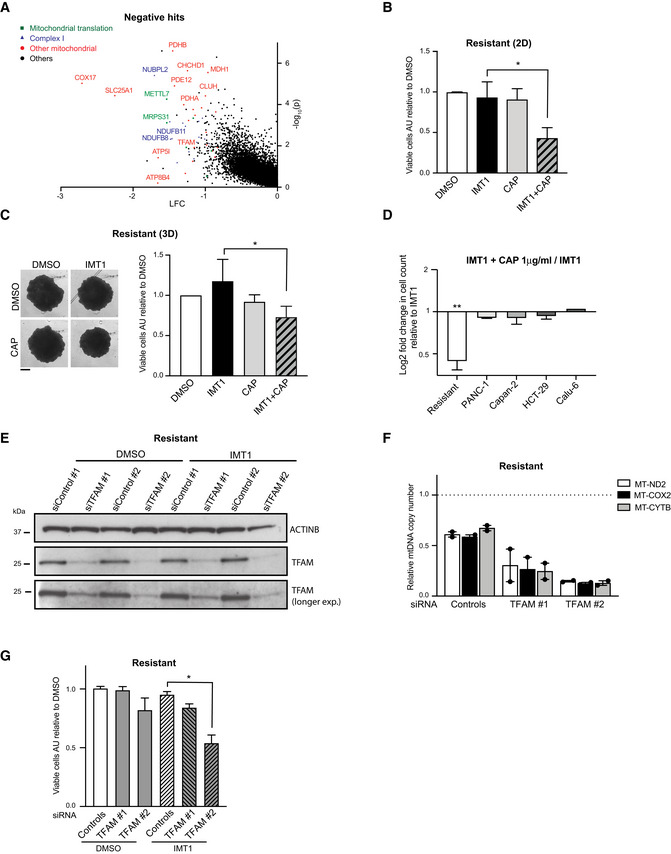
Inhibition of mitochondrial translation and decrease in mitochondrial DNA (mtDNA) copy number sensitize resistant cells to IMT1 treatment Depleted CRISPR (clustered regularly interspaced short palindromic repeat) guides (negative hits) in IMT1‐treated RKO cells plotted as log_2_‐fold change (LFC) versus log_10_ of adjusted *P*‐value.Viability assessment of monolayer cultures of IMT1‐resistant RKO cells treated for 1 week with either dimethyl sulfoxide (DMSO), IMT1 (1 µM), chloramphenicol (CAP) (1 µg/ml) alone or in combination with IMT1. Data are expressed as mean values ± SD of *n* = 4 independent experiments, each including four technical intra‐plate replicates. Statistical significance was calculated with one‐way ANOVA test. IMT1 versus IMT1+ CAP: *P = 0.0301.Representative images and related plot of IMT1‐resistant RKO spheroid areas in the presence of the aforementioned compounds for 2 weeks (IMT1: 1 µM, CAP: 1 µg/ml); scale bar: 1 mm. Data show the mean values ± SD of *n* = 4 experiments. Statistical significance was calculated with paired t‐test: IMT1 versus IMT1+ CAP: *P = 0.0242.Log_2_‐fold changes (LFCs) in viable cell count in a panel of five IMT1‐resistant cancer cell lines in the presence of IMT1 + CAP (chloramphenicol) (1 µg/ml). Data represent mean values of *n* = 5 (resistant RKO cells) and *n* = 3 (PANC‐1, Capan‐2, HCT‐29, and Calu‐6) ± SD independent experiments. Paired t‐test of IMT1 versus IMT1+CAP: resistant RKO cells **P = 0.0014; PANC‐1, Capan‐2, HCT‐29, and Calu‐6: non‐significant.Western blot analyses of mitochondrial transcription factor A (TFAM) protein’s steady‐state levels in IMT1‐resistant RKO cells in the presence of small interfering RNAs (siRNAs) against controls (control #1, #2) and TFAM (TFAM #1, TFAM #2) and treated with dimethyl sulfoxide (DMSO) or IMT1. Images are representative of *n* = 3 independent experiments. Actin B (ACTINB) is shown as loading control.Mitochondrial DNA (mtDNA) levels in IMT1‐treated resistant RKO cells in the presence of small interfering (siRNA) against controls (control #1, #2) or mitochondrial transcription factor A (TFAM) (TFAM #1, TFAM #2). Data are expressed as relative to DMSO‐treated resistant RKO cells treated with control siRNAs (dotted line = 1) and show the average of *n* = 2 independent experiments.Viable cell counts of IMT1‐resistant RKO cells treated for 1 week with either dimethyl sulfoxide (DMSO) or IMT1 in the presence of controls (control #1, #2) or mitochondrial transcription factor A (TFAM) (TFAM #1, TFAM #2) small interfering RNAs (siRNAs). Data are expressed as mean values ± SD of *n* = 5 independent experiments, each including six technical intra‐plate replicates. Statistical significance was calculated with one‐way ANOVA test. Controls + IMT1 versus TFAM #1 + IMT1: *P* = 0.2148; Controls + IMT1 versus TFAM #2 + IMT1: *P = 0.0415.

The CRISPR‐Cas9 screen, in addition to positive hits (Fig 1F), also identified a number of genes whose inactivation increased the cellular sensitivity to IMT1 (negative hits), for example, genes encoding OXPHOS subunits, the mitochondrial transcription factor A (TFAM), and several mitochondrial translation factors (Fig 7A and Dataset EV2). To test the role of mitochondrial translation further, we treated cells with chloramphenicol (CAP), a well‐known inhibitor of mitochondrial translation (McKee *et al*, 2006). We performed a dose–response titration in monolayer cultures of RKO cells and found that CAP concentrations below 1 µg/ml did not cause cell death (Fig EV4A), despite decreasing the levels of the mtDNA‐encoded COX2 protein (Fig EV4B). Next, we proceeded to treat RKO cells and IMT1‐resistant RKO cells with this sublethal dose of CAP (1 µg/ml) and found no effect on cell viability (Figs EV4C and 7B). Although the IMT1‐resistant cells showed no response to IMT1 and CAP when administered individually, the combined treatment with both drugs caused a 50% drop in cell viability in monolayers of cells (Fig 7B), showing an additive effect of the two drugs. Importantly, IMT1 and CAP co‐treatment did not affect the viability of primary fibroblasts from healthy donors (Fig EV4D). We then proceeded to analyze spheroids, as this culture system resembles better some aspects of tumor biology than monolayer cultures (Minchinton & Tannock, 2006). We treated spheroids of RKO cells with CAP and found that doses above 1 µg/ml impaired their growth (Fig EV4E). When we treated RKO cells with a combination of IMT1 and CAP in increasing concentrations, we found an additive effect on spheroidal growth (Fig EV4F). Next, we treated spheroids of IMT1‐resistant RKO cells with IMT1 and CAP and found a similar effect (Fig 7C). To understand whether the CAP could sensitize other IMT1‐resistant cell lines to IMT1 treatment, we tested this treatment on four additional cell lines. We found that 1 µg/ml of CAP mildly reduced viability in the presence of IMT1 in three of the four IMT1‐resistant cell lines tested (Fig 7D). When the concentration of CAP was raised to 100 µg/ml, the drop in cell number was much stronger (Fig EV4G). We also tested whether the inhibition of glycolysis can sensitize IMT‐resistant cell lines to IMT1 treatment, but found only a mild difference in viability when a combination of 1 mM 2DG and IMT1 was administered (Fig EV4H).

Since the IMT1‐resistant cells maintained higher levels of mtDNA in the presence of IMT1 (Fig 4G), we investigated whether decreased mtDNA levels could affect survival. To this end, we knocked down the expression of TFAM, which directly controls mtDNA levels (Filograna *et al*, 2021; Bonekamp *et al*, 2021). We used two different small interfering RNAs (siRNAs) in IMT1‐resistant cells and both siRNAs caused a gradual decrease in TFAM protein levels (Fig EV4I) and mtDNA levels (Fig EV4J) over time. TFAM siRNA#2 caused a stronger decrease of TFAM protein levels and mtDNA copy number than TFAM siRNA#1 (Fig 7E and F). Consistently, there was a significant decrease in cell viability when IMT1‐resistant cells were transfected with TFAM siRNA#2 in comparison with cells transfected with control siRNAs, in the presence of IMT1 (Fig 7G). The importance of TFAM in preventing IMT1 toxicity is also supported by the CRISPR‐Cas9 screen where TFAM was identified as a negative hit (Fig 7A).

## Discussion

In this study, we used IMT1, a highly specific allosteric inhibitor of POLRMT (Bonekamp *et al*, 2020), to study resistance mechanisms in treated cancer cells. We used an unbiased whole‐genome CRISPR‐Cas9 screen to identify resistance mechanisms induced by acute IMT1 treatment and found that the loss of mTORC1 and VHL expression was protective. The mTORC1 protein complex functions as a nutrient sensor and controls cytosolic translation, which makes it one of the master regulators of cell proliferation and cell fate decisions. Inhibitors of mTORC1 have been proposed for cancer treatment (Podsypanina *et al*, 2001; Thimmaiah *et al*, 2010), but the outcomes from clinical studies have not been conclusive (Sun, 2013). It has also been proposed that mTORC1 activation may occur although nutrients are scarce in cancer cells and that rapamycin treatment can be protective and prevent cell death (Fumarola *et al*, 2005; Choo *et al*, 2010; Hung *et al*, 2012; Villar *et al*, 2017). The data we present here support this model because the decreased cell viability caused by IMT1 treatment was rescued by rapamycin. Consistent with these results, rapamycin treatment has also been reported to slow disease progression and prolong survival in mice with mitochondrial dysfunction (Zheng *et al*, 2016; Khan *et al*, 2017).

When oxygen levels are low, the stabilization of HIF1α induces a transcriptional reprogramming of the cellular metabolism to decrease the dependency on mitochondrial function (Papandreou *et al*, 2006; Zhang *et al*, 2007). Loss of VHL, which causes oxygen‐independent stabilization of HIF1α, is reported to increase the predisposition to some cancers (Giles *et al*, 2006; Kaelin, 2008). The PHD inhibitor FG4592 (Roxadustat) is routinely used *in vitro* as an experimental tool to stabilize HIF1α (Jain *et al*, 2016) and was under FDA evaluation for anemia treatment (https://clinicaltrials.gov/ct2/show/NCT01750190; Guenzler‐Pukall *et al*, 2003; Rabinowitz, 2013; Joharapurkar *et al*, 2018). Our results show that FG4592 stabilizes HIF1α and protects against cell death induced by IMT1, which is in good agreement with published data showing that loss of VHL or FG4592 treatment rescues the effects of toxins that inhibit OXPHOS (Jain *et al*, 2016). It has been reported that hypoxia can prevent neurodegeneration in a mouse model with mitochondrial dysfunction (Jain *et al*, 2016; Ferrari *et al*, 2017). However, stabilization of HIF1α by genetic interventions was not sufficient to prevent neurodegeneration in this model (Jain *et al*, 2019), where complex I becomes unstable due to the absence of the NADH dehydrogenase (ubiquinone) iron‐sulfur protein 4 (NDUFS4) subunit (Sterky *et al*, 2012). The pathology was instead attributed to a direct effect of high oxygen tension in the brain (Jain *et al*, 2019). In contrast, HIF1α has a direct role in ameliorating the acute effects of impaired mtDNA expression in IMT1‐treated tumor cells. It is possible that HIF1α expression is of importance in the large subgroup of mitochondrial diseases where the biogenesis of the whole OXPHOS system is impaired, whereas it may have no role in mitochondrial diseases caused by impaired stability of a single complex.

Alterations of cellular metabolism and mitochondrial function are well‐known hallmarks of cancer (Hanahan & Weinberg, 2011). In contrast to many specialized postmitotic cells, cancer cells do not only need mitochondria to sustain ATP production, but also to supply a variety of metabolic intermediates and reducing equivalents needed for synthesis of biomass during cellular proliferation (Vander Heiden *et al*, 2009; Vasan *et al*, 2020). Mitochondria are thus an emerging target for cancer treatment, but the resistance mechanisms induced by chronic inhibition of mitochondrial function are poorly understood. To investigate the effects of chronic IMT1 resistance, we used a dose‐escalation approach in RKO cells. The IMT1‐resistant RKO cells maintained mtDNA expression at higher levels than RKO cells, and this was sufficient to increase the levels of cellular metabolites. However, interventions to decrease mtDNA copy number (TFAM knockdown) or to impair mitochondrial translation (CAP treatment) decreased the survival of the IMT1‐treated resistant cells. These findings argue that interventions at different levels in the mtDNA expression axis, for example, decrease of mtDNA copy number, mitochondrial transcription, or mitochondrial translation, act synergistically. A threshold effect seems to be important for cancer cell survival in both the acute and chronic IMT1‐induced resistance. In fact, both rapamycin and chronic IMT1 treatments seem to confer resistance by maintaining mtDNA‐encoded gene products at higher levels. Also, inhibitors of mitochondrial translation, such as the FDA‐approved drug tigecycline (Jia *et al*, 2016; Kuntz *et al*, 2017), have been effective in preclinical tumor models. Although the exact role of mtDNA level variation in cancer is not fully understood, an upregulation of mtDNA copy number commonly occurs in many tumors (Reznik *et al*, 2016; Filograna *et al*, 2021; Yuan *et al*, 2020). It is important to note that studies of humans (Larsson & Clayton, 1995; Stewart & Chinnery, 2015) and mice (Kauppila *et al*, 2016; Jiang *et al*, 2017; Filograna *et al*, 2019) with mitochondrial dysfunction have shown that mtDNA expression below a critical threshold level will lead to a cellular energy crisis. This threshold phenomenon likely explains why a modest increase of cellular respiration can drastically increase the levels of cellular metabolites and survival of cancer cells, as observed here.

Due to the extreme genetic heterogeneity of cancer cell lines, it is not surprising that responses to the inhibition of mitochondrial gene expression are not universal. However, we have identified a number of mechanisms that appear to be common to many cancer cell lines. The responses identified in this study should therefore be taken into consideration when future mitochondria‐targeted therapies are developed.

## Materials and Methods

### Cell lines, cell culture conditions, and treatments

RKO (ATCC, CRL‐2577), MiaPaCa‐2 (ATCC, CRL‐1420), HeLa (ATCC, CCL2), Calu‐6 (ATCC, HTB‐56), NCIH‐460 (ATCC, HTB‐177), DLD‐1 (ATCC, CLL‐221), HT‐29 (ATCC, HTB‐38), Capan‐2 (ATCC, HTB‐80), PANC‐1 (ATCC, CRL‐1469), A549 (DSMZ, ACC107), and HCT‐15 (DSMZ, ACC‐357) cells and primary fibroblasts were all maintained in Dulbecco’s modified Eagle’s medium (DMEM) GlutaMAX^TM^ (Gibco, 31966‐021) supplemented with 10% fetal bovine serum (FBS, Gibco 10270‐106), 1% penicillin and streptomycin (Gibco, 15140122) at 37°C in a 5% CO_2_ atmosphere. Anonymized human primary fibroblasts used in this study were obtained from the Center for Inherited Metabolic Diseases (CMMS) at the Karolinska University Hospital. The samples were obtained in accordance with the Declaration of Helsinki, the Department of Health and Human Services Belmont Report, and approved by the Regional Ethics Committee at Karolinska Institutet in Stockholm, Sweden. After titration, the working concentrations for the treatments were selected as follows: IMT1 1 µM (LDC195943, LDC Discovery Center (Bonekamp *et al*, 2020)), Chloramphenicol 1 µg/ml (Sigma, C0378), Rapamycin 100 nM (Life Technologies, PHZ1235) and Temsirolimus 100 nM (Sigma, PZ0020), FG4592 100 µM (MedChemExpress, HY‐13426/CS1094), and 2DG 1 mM (Sigma, D8375). To estimate the cellular autophagic flux, at the end of each treatment, vehicle (water) or NH_4_Cl (20 mM) was added to each well for 3 h to inhibit lysosomal acidification before the collection of the cells.

### TFAM downregulation

Downregulation experiments were performed using two different Silencer Select siRNAs for controls or TFAM according to the manufacturer’s specifications (Thermo Fisher, Silencer Select, Control#1, 4390843, Control #2, 4390846, TFAM#1, 4392420 – s14002, TFAM#2, 4392420 – s14000). Cells were assessed at 24, 48, and 96 h after silencing to identify changes in TFAM protein levels and mtDNA copy number. For studying the effect of TFAM downregulation on viability, the silencing was performed in a 96‐well format 24 h after seeding and the treatment with DMSO or IMT1 was started 48 h after seeding. A second round of transfection was performed at 96 h from seeding and viable cells were counted after 6 days of treatment, as described in the next section.

### Viability assessment

The viability assays for 2D cultures were performed in a 96‐well format as follows: 1,000 cells per well were plated and the treatments were started 24 h after seeding. The length of each treatment is specified in the Figures section for all the experiments. Cellular viability was determined using Cell Counting Kit 8 (Sigma, 96992), which selectively stains viable cells. IC_50_ was determined by non‐linear least squares fit of inhibitor concentration logarithm against cell count in GraphPad Prism 9 software. For 3D cultures, 500 cells per well were plated in round bottom 96‐well plates (Corning, 7007). After 2 weeks of treatment, the bright field images of the cells were obtained and analyzed with the software Fiji (ImageJ) to determine the diameter of the spheroids.

### Generation of IMT1‐resistant RKO cells

Pool populations of RKO cells were subjected to dose‐escalated treatments with IMT1. The cells were treated with increasing concentrations of the compound over several months, starting with a sublethal concentration of 10 nM for the first month, increased to 100 nM during the second, raised to and maintained at 1 µM during the following 4 months. The cells showed to adapt and survive to all IMT1 doses and to maintain the resistance phenotype over freeze‐thaw cycles. Two independent batches of resistant cells were generated with the described approach and both showed to behave in the same way in respect to the parameters analyzed in this study.

### Northern blotting and quantitative real‐time PCR

The total RNA was extracted from cell pellets using TRIzol reagent (Life Technologies, 15596018) and spectrophotometrically quantified by measuring the absorbance at 260 nm. For quantification of the transcripts, the RNA was retrotranscribed using the High‐Capacity cDNA Reverse Transcription Kit (Applied Biosystems, 4368814) and the relative complementary DNAs (cDNAs) were measured by the quantitative real‐time polymerase chain reaction (qRT–PCR) using TaqMan Universal Master Mix II and probes (Applied Biosystems, 442873) and normalized to Actin B (ACTINB) cDNA. The assays were performed in technical triplicates on 384‐well reaction plates (Applied Biosystems) in final volumes of 10 µl and according to the manufacturer’s guidelines. Transcript half‐lives were determined at time curves of 0, 0.5, 1, 2, 4, 6, 24, 48, 72, and 96 h. Half‐lives of mitochondrial transcripts were estimated by non‐linear regression curve fitting using GraphPad Prism 5.0 software.

For northern blotting, 4 µg of extracted RNA was run on an agarose (1.2%) formaldehyde (18%) gel in 3‐(N‐morpholino) propanesulfonic acid (MOPS) buffer (Ambion), then transferred to Hybond‐N+ nylon membranes (GE Healthcare) and hybridized with α‐^32^P‐CTP‐labeled probes (PerkinElmer) for mitochondrial‐encoded RNA species. The probes were generated using the 9‐mer random primer kit, according to the manufacturer’s specifications (Agilent).

### DNA extraction, mtDNA copy number, and POLRMT sequencing

Total DNA was extracted from cell pellets using QIAmp DNA extraction kit (Qiagen) according to the manufacturer’s specifications. Total DNA levels were measured and, after a ribonuclease (RNAse) digestion step, the mtDNA copy number was measured via qRT–PCR using TaqMan probes against ND1, ATP6, and COX2 and normalized using 18S nuclear DNA. The assays were performed in technical triplicates on 384‐well reaction plates (Applied Biosystems) in final volumes of 10 µl and according to the manufacturer’s guidelines. Sanger sequencing of human POLRMT gene was performed in parental and resistant RKO cells in samples belonging to three independent experiments using two different primer pairs to amplify the region of interest. Primers were designed as follows: F1_CTACCGTCAAGGGTTGGTCC; R1_TGGTGCTGCAGCTCTTCC; and F2_TCAAC CCCGTGAGATTGACC; R2_GTGCTGCAGCTCTTCCAGG).

### Mitochondrial translation assay

^35^S‐labeling was performed, as previously described (Chomyn, 1996). Briefly, after washes with cysteine‐ and methionine‐free DMEM (Gibco, 21013024), the cells were incubated in the same medium supplemented with 1 mM glutamax, 1 mM sodium pyruvate (Life Technologies), and 10% dialyzed bovine serum. As much as 100 μg/ml of anisomycin (Sigma, A9789) was added for 20 min to inhibit the cytosolic protein synthesis and 3.7 MBq of ^35^S‐L‐methionine and cysteine mix (PerkinElmer, NEG77200) was added for 45 min to label the newly synthesized mitochondrial proteins. After harvesting, the cells were lysed on ice in phosphate‐buffered saline solution (PBS) containing protease inhibitor cocktail (Roche, 4693116001), benzonase nuclease (Sigma, E1014 (2.25 U/μl)), and 0.1% n‐dodecyl‐β‐D‐maltoside (DDM) (Sigma, D4641). One percent SDS was then added to complete the lysis. For the chase experiment, cells were pre‐treated with 50 μg/ml of chloramphenicol for 16 h before the experiment and collected 24 h after the ^35^S‐labeling. Proteins (20–30 μg) per lane were loaded on pre‐cast SDS–PAGE gels (Life Technologies). The gels were stained with Coomassie staining solution (50% methanol, 10% acetic acid, and 0.1% Coomassie Brilliant Blue R250 (Thermo Fisher, 20278)) to ensure equal loading. The gels were dried and exposed to phosphor screens and the signal was detected using Typhoon^TM^ Phosphoimager. Densitometric quantification of band intensity was performed using the software Fiji and normalized for Coomassie‐stained images of the gels.

### Western blotting

After harvesting, the cell pellets were resuspended in PBS and lysed on ice with lysis buffer (4 mM Tris–HCL, 1 M NaCl, 4 mM ethylenediaminetetraacetic acid (EDTA, Sigma), 2% Triton X‐100 (Santa Cruz Biotechnology), 20% glycerol, 1× protease inhibitor cocktail, phosphatase inhibitor cocktail (Sigma, 4906845001), and 2.25 U/µl benzonase). After incubating on ice for 20 min, the samples were centrifuged for 20 min at 13,000 *g*, and the protein‐containing supernatant was used for loading. Equal amounts of proteins were separated on NuPAGE gels (Life Technologies) depending on the separation profile needed. Proteins were transferred to 0.45‐μm polyvinylidene fluoride membranes (PVDF, Millipore IPVH00010) and blocked in 5% milk Tris‐buffered saline (TBS) solution containing 0.1% Tween for 1 h. The membranes were incubated with primary antibodies overnight at 4°C (Actin B, Abcam (ab8226); human total OXPHOS cocktail, Abcam (ab110411); glyceraldehyde 3‐phosphate dehydrogenase (GAPDH), Abcam (ab8245); 4EBP1 total, Cell Signaling (9644); phospho‐4EBP1 (S65), Cell Signaling (9451); HIF1‐α, Cell Signaling (14179); VHL, Cell Signaling (68547), TFAM, Abcam (ab131607), and LC3B Cell Signaling (#2775)). After washes in TBS‐Tween, the membranes were incubated with anti‐mouse or anti‐rabbit secondary antibodies (GE Healthcare, NA9310V and NA9340V) for 1 h at room temperature. Proteins were detected using Clarity Western ECL Substrate (Bio‐Rad, 170‐5061). Densitometric quantification of western blot bands was performed using the software Fiji normalized for the loading control.

### Oxygen consumption and extracellular acidification rates’ measurement

Parental and resistant RKO cells were seeded in Seahorse XFe96 culture dishes at a density of 1,000 cells per well and treatments were started 24 h after seeding. OCR and ECAR were measured simultaneously on a Seahorse XFe96 analyzer (Agilent Technologies) in unbuffered DMEM containing glucose (10 mM), glutamine (2 mM), pyruvate (2 mM), and Hepes (5 mM). After assessment of basal OCR and ECAR rates, sequential addition of oligomycin (oligo, 1 μM), carbonyl cyanide‐*p*‐(trifluoromethoxy)phenylhydrazone (FCCP, 0.25 μM), rotenone/antimycin A (Rot, 1 μM/ AA, 1 μM), and 2‐deoxy‐D‐glucose (2DG, 50 mM) was performed. The optimal concentrations of these metabolic modulators were investigated in pre‐experiments. Three independent experiments with at least six replicates were performed. After each experiment, OCR and ECAR values were normalized to corresponding cell numbers in each well using the CyQUANT assay (Thermo Fisher). Steady states of basal and maximal OCR or ECAR were calculated as the mean of three measurement cycles and were corrected for non‐mitochondrial OCR (after Rot/AA addition) or non‐glycolytic ECAR (after 2DG addition). Maximal OCR and ECAR corresponded to the steady states after injection of the ATP‐synthase inhibitor oligomycin and uncoupler FCCP.

### Protein extraction, proteolytic digestion, and chemical labeling

Cell pellets were suspended in 0.1% ProteaseMAX (Promega), 4 M urea (Sigma‐Aldrich), 50 mM ammonium bicarbonate, and 10% acetonitrile (ACN). The samples were sonicated using Vibra‐Cell probe (Sonics & Materials, Inc.) for 1 min, with pulse 2/2, at 20% amplitude, and sonicated in bath for 5 min, followed by vortexing and centrifugation for 5 min at 20,000 *g*. The supernatants were transferred to new tubes and the concentration was determined in a 1:10 dilution, in water. The protein yields were 500–1,300 µg.

Twenty‐five micrograms (25 µg) of each sample was subjected to a tryptic digestion protocol including protein reduction in 6 mM dithiothreitol at 37°C for 60 min and alkylation in 22 mM iodoacetamide for 30 min at room temperature in the dark. Trypsin was added in an enzyme‐to‐protein ratio of 1:50 and digestion was carried out overnight at 37°C. Tryptic peptides were cleaned with C18 HyperSep Filter Plate, bed volume 40 µl (Thermo Scientific) and dried on a speedvac (miVac, Thermo Scientific). Six of TMT‐10plex reagents (Thermo Scientific) in 100‐µg aliquots were dissolved in 30‐µl dry AcN, scrambled and mixed with the digested samples dissolved in 70 µl of triethylammonium bicarbonate (TEAB) (resulting final 30% AcN), followed by incubation at 22°C for 2 h at 550 rpm. The reaction mixture was then quenched with 12 µl of 5% hydroxylamine at 22°C for 15 min at 550 rpm. The labeled samples were pooled and dried on a speedvac (miVac, Thermo Scientific).

### Liquid chromatography tandem mass spectrometry

Twenty micrograms of tandem mass tag (TMT)‐labeled tryptic peptides was dissolved in 20 µl of 2% ACN/0.1% formic acid. Two µg samples were injected into a nano LC‐1000 system online coupled to an Orbitrap Fusion mass spectrometer (Thermo Scientific, Bremen, Germany). The chromatographic separation of the peptides was achieved using a 50‐cm long C18 EASY‐spray column (Thermo Scientific), with the following gradient: 2–26% ACN in 110 min, 26–35% ACN in 10 min, 35–95% ACN in 5 min, and 95% ACN for 15 min at a flow rate of 300 nl/min. The MS acquisition method was comprised of one survey full scan ranging from *m/z* 375 to 1,500, acquired with a resolution of R = 120,000 (at *m/z* 200), followed by data‐dependent higher‐energy C‐trap dissociation (HCD) fragmentations from maximum 15 most intense precursor ions with charge states 2+ and 7+. The tandem mass scans were acquired with a resolution of R = 60,000, targeting 5 × 10^4^ ions, setting isolation width to *m/z* 1.4, and normalized collision energy to 35%.

### Proteomic data analysis

The raw data files were directly loaded in Proteome Discoverer v2.2 and searched against mouse or human SwissProt protein databases (42,793 and 21,008 entries, respectively) using the Mascot 2.5.1 search engine (Matrix Science Ltd.). Parameters were chosen as follows: up to two missed cleavage sites for trypsin, precursor mass tolerance 10 ppm, and 0.05 Da for the HCD fragment ions. Dynamic modifications of oxidation on methionine, deamidation of asparagine and glutamine, and acetylation of N‐termini were set. For quantification, both unique and razor peptides were requested. The final quantitative data analysis was performed with an in‐house developed R‐studio script. Submitochondrial localization was based on the data extracted from (Vögtle *et al*, 2017). Human homologs were mapped with DIOPT v.8.0 (Hu *et al*, 2011) and filtered for proteins in human MitoCarta 2.0 (Calvo *et al*, 2016).

### Measurement of intracellular IMT1 concentration

Parental and resistant RKO cells were seeded in triplicates in 12‐well dishes at a density of 0.5 × 10^6^ cells per well in phenol‐red free complete DMEM (Gibco 31053‐028). Twenty‐four hours after seeding, parental RKO cells were treated with either DMSO or 1 µM IMT1. IMT1‐resistant cells were continuously grown in 1 µM IMT1. Samples were collected 0, 2, or 24 h after treatment, and the supernatant was transferred and snap‐frozen. The cells were washed, acetonitrile (ACN) was added and incubated for 15 min at 4°C for cell extraction. The ACN extracts were transferred to a test tube and snap‐frozen. Samples were analyzed by liquid chromatography–tandem mass spectrometry (LC–MS) using a Prominence UFLC system (Shimadzu) coupled to a Qtrap 5500 instrument (ABSciex). Prior to analysis, the samples were extracted with ACN containing internal standard, filtered, and diluted with water as necessary. The analytes were separated on a C18 column with an ACN and water mixture containing 0.1% formic acid as solvent using a gradient elution. Multiple reaction monitoring (MRM) transitions for each analyte were optimized automatically. Test article concentrations were calculated using a standard curve.

### CRISPR‐Cas 9 screening

The human colon carcinoma cell line RKO (ATCC^®^ CRL‐2577™) was first made to stably express the Cas9 nuclease as described in (Schmierer *et al*, 2017). In brief, a construct coding for Cas9, blasticidin resistance, and a single guide against hypoxanthine phosphoribosyltransferase 1 (HPRT1) was introduced with lentivirus transduction. This allows sequential selection with blasticidin and 6‐thioguanine, a nucleotide analog lethal in HPRT1^+^ cells. Next, two replicates of approximately 150 M cells each were transduced with the genome‐wide Brunello sgRNA library (Doench *et al*, 2016) at an MOI (multiplicity of infection) of 0.4 (> 600 cells/guide) in 2 µg/ml of polybrene. Transduced cells were selected with puromycin (1 µg/ml) from post‐transduction days 2–7 and then allowed to grow for an additional 4 days without puromycin. Cells were subcultured when necessary, with cell number never dropping below 75 M. Cells were subcultured on post‐transduction day 11, and on day 12 populations of 150 M cells were treated with either DMSO or 1 µM IMT1. After 10 days of treatment during which DMSO‐treated cells were subcultured every 2–3 days and inhibitor‐treated cells were subcultured once, cells were harvested, the DNA was isolated (Qiagen DNeasy Blood & Tissue kit), and guide sequences were amplified by PCR, as described in Schmierer *et al* (2017). Pathway analysis performed using Reactome database (Jassal *et al*, 2019) on the top 50 significant enriched or depleted hits identified mTORC1 pathway as positive hit. Whereas, VHL gene was the most enriched and significant positive hit, whose loss rescued IMT1 toxicity.

### Metabolite extraction for liquid chromatography mass spectrometry

RKO wild‐type and IMT1‐resistant cells were seeded into 6‐well cell culture dishes (1 × 10^6^ cells per well). Twenty‐four hours after seeding, the RKO cells were treated with either DMSO or 1 μM IMT1 for 96 h. Resistant RKO cells were kept in the continuous presence of 1 µM IMT1. Metabolites were extracted from parental and resistant RKO cells to determine basal differences in metabolite levels (0 h). After removal of the growth medium, the cells were washed twice with ammonium carbonate buffer (75 mM, pH 7.4) at 37°C. Metabolites were then extracted by two consecutive incubations with extraction buffer (40/40/20 (v/v/v) ACN/methanol/water, 10 ng/ml ^13^C_10_ATP, citric acid d4 (Sigma 710695, 485438), as well as 2.5 nM uniformly ^13^C^15^N‐labeled amino acids (Cambridge Isotope Laboratories, MSK‐A2‐1.2)). The two extracts were pooled and centrifuged. The supernatants were transferred to new tubes and dried in a speed vac concentrator (Eppendorf). Samples were resuspended in 150 µl of liquid chromatography mass spectrometry (LC–MS)‐grade H_2_O (Thermo Fisher Scientific) of which, one aliquot (50 µl) was used to perform anion‐exchange chromatography for the analysis of glycolysis, TCA, nucleotide and deoxy nucleotide metabolites, and another aliquot (50 µl) was used for the analysis of amine‐containing metabolites. LC–MS analysis of amine‐containing metabolites and anion‐exchange chromatography mass spectrometry for the analysis of nucleotides, TCA cycle and glycolysis metabolites were performed, as previously described in Bonekamp *et al* (2020). Each peak was normalized to the intensities of the appropriate internal standard that were spiked into the extraction buffer. Additionally, the peak intensities were normalized for the sample amount equivalent, hence the obtained protein concentration of each sample.

### Statistical analysis

Data are expressed as mean values ± standard deviation (SD). Group mean values were analyzed using a two‐tailed non‐parametric Student’s *t*‐test, whereas multiple comparisons were performed with one‐way ANOVA test. Comparisons were considered statistically significant for *P* values < 0.05 (*P < 0.05, **P < 0.005, ***P < 0.001, ****P < 0.0001).

## Author contributions

MM and N‐GL conceived the project, designed the experiments, and wrote the manuscript. MM performed the majority of the experiments. RF, AF, NAB, and OL performed experiments and analyzed the data generated. PG performed the metabolomics analyses. N‐GL supervised the project. All authors gave input on the manuscript.

## Conflict of interest

The IMT1 compound intellectual property is published as WO 2019/057821 and has been licensed by the Max Planck Society and the Lead Discovery Center GmbH. NGL is a scientific founder and holds stock in Pretzel Therapeutics, Inc.

## Supporting information



Expanded View Figures PDF

Dataset EV1

Dataset EV2

Dataset EV3

## Data Availability

The mass spectrometry proteomics data have been deposited to the ProteomeXchange Consortium via Proteomics Identification Database (PRIDE) partner repository (Perez‐Riverol *et al*, 2019) with the dataset identifier PXD026481 and will be available at the following link: https://www.ebi.ac.uk/pride/archive/projects/PXD026481

## References

[embr202153054-bib-0001] Anderson S , Bankier AT , Barrell BG , de Bruijn MHL , Coulson AR , Drouin J , Eperon IC , Nierlich DP , Roe BA , Sanger F *et al* (1981) Sequence and organization of the human mitochondrial genome. Nature 290: 457–465 7219534 10.1038/290457a0

[embr202153054-bib-0002] Bibb MJ , Van Etten RA , Wright CT , Walberg MW , Clayton DA (1981) Sequence and gene organization of mouse mitochondrial DNA. Cell 26: 167–180 7332926 10.1016/0092-8674(81)90300-7

[embr202153054-bib-0003] Bonekamp NA , Jiang M , Motori E , Villegas RG , Koolmeister C , Atanassov I , Mesaros A , Park CB , Larsson N‐G (2021) High levels of TFAM repress mammalian mitochondrial DNA transcription *in vivo* . Life Sci Alliance 4: e202101034 34462320 10.26508/lsa.202101034 PMC8408345

[embr202153054-bib-0004] Bonekamp NA , Peter B , Hillen HS , Felser A , Bergbrede T , Choidas A , Horn M , Unger A , Di Lucrezia R , Atanassov I *et al* (2020) Small‐molecule inhibitors of human mitochondrial DNA transcription. Nature 588: 712–716 33328633 10.1038/s41586-020-03048-z

[embr202153054-bib-0005] Boukalova S , Stursa J , Werner L , Ezrova Z , Cerny J , Bezawork‐Geleta A , Pecinova A , Dong L , Drahota Z , Neuzil J (2016) Mitochondrial targeting of metformin enhances its activity against pancreatic cancer. Mol Cancer Ther 15: 2875–2886 27765848 10.1158/1535-7163.MCT-15-1021

[embr202153054-bib-0006] Calvo SE , Clauser KR , Mootha VK (2016) MitoCarta2.0: an updated inventory of mammalian mitochondrial proteins. Nucleic Acids Res 44: D1251–D1257 26450961 10.1093/nar/gkv1003 PMC4702768

[embr202153054-bib-0007] Chinnery PF (2015) Mitochondrial disease in adults: what’s old and what’s new? EMBO Mol Med 7: 1503–1512 26612854 10.15252/emmm.201505079 PMC4693502

[embr202153054-bib-0008] Chomyn A (1996) *In vivo* labeling and analysis of human mitochondrial translation products. Methods Enzymol 264: 197–211 8965693 10.1016/s0076-6879(96)64020-8

[embr202153054-bib-0009] Choo AY , Kim SG , Vander Heiden MG , Mahoney SJ , Vu H , Yoon S‐O , Cantley LC , Blenis J (2010) Glucose addiction of TSC null cells is caused by failed mTORC1‐dependent balancing of metabolic demand with supply. Mol Cell 38: 487–499 20513425 10.1016/j.molcel.2010.05.007 PMC2896794

[embr202153054-bib-0010] Doench JG , Fusi N , Sullender M , Hegde M , Vaimberg EW , Donovan KF , Smith I , Tothova Z , Wilen C , Orchard R *et al* (2016) Optimized sgRNA design to maximize activity and minimize off‐target effects of CRISPR‐Cas9. Nat Biotechnol 34: 184–191 26780180 10.1038/nbt.3437 PMC4744125

[embr202153054-bib-0011] Ferrari M , Jain IH , Goldberger O , Rezoagli E , Thoonen R , Cheng K‐H , Sosnovik DE , Scherrer‐Crosbie M , Mootha VK , Zapol WM (2017) Hypoxia treatment reverses neurodegenerative disease in a mouse model of Leigh syndrome. Proc Natl Acad Sci USA 114: E4241–E4250 28483998 10.1073/pnas.1621511114 PMC5448167

[embr202153054-bib-0012] Filograna R , Koolmeister C , Upadhyay M , Pajak A , Clemente P , Wibom R , Simard ML , Wredenberg A , Freyer C , Stewart JB *et al* (2019) Modulation of mtDNA copy number ameliorates the pathological consequences of a heteroplasmic mtDNA mutation in the mouse. Sci Adv 5: eaav9824 30949583 10.1126/sciadv.aav9824 PMC6447380

[embr202153054-bib-0013] Filograna R , Mennuni M , Alsina D , Larsson N‐G (2021) Mitochondrial DNA copy number in human disease: the more the better? FEBS Lett 595: 976–1002 33314045 10.1002/1873-3468.14021 PMC8247411

[embr202153054-bib-0014] Fumarola C , La Monica S , Alfieri RR , Borra E , Guidotti GG (2005) Cell size reduction induced by inhibition of the mTOR/S6K‐signaling pathway protects Jurkat cells from apoptosis. Cell Death Differ 12: 1344–1357 15905878 10.1038/sj.cdd.4401660

[embr202153054-bib-0015] Funes JM , Quintero M , Henderson S , Martinez D , Qureshi U , Westwood C , Clements MO , Bourboulia D , Pedley RB , Moncada S *et al* (2007) Transformation of human mesenchymal stem cells increases their dependency on oxidative phosphorylation for energy production. Proc Natl Acad Sci USA 104: 6223–6228 17384149 10.1073/pnas.0700690104 PMC1851087

[embr202153054-bib-0016] Gammage PA , Frezza C (2019) Mitochondrial DNA: the overlooked oncogenome? BMC Biol 17: 53 31286943 10.1186/s12915-019-0668-y PMC6615100

[embr202153054-bib-0017] Giles RH , Lolkema MP , Snijckers CM , Belderbos M , van der Groep P , Mans DA , van Beest M , van Noort M , Goldschmeding R , van Diest PJ *et al* (2006) Interplay between VHL/HIF1alpha and Wnt/beta‐catenin pathways during colorectal tumorigenesis. Oncogene 25: 3065–3070 16407833 10.1038/sj.onc.1209330

[embr202153054-bib-0018] Gopal RK , Calvo SE , Shih AR , Chaves FL , McGuone D , Mick E , Pierce KA , Li Y , Garofalo A , Van Allen EM *et al* (2018) Early loss of mitochondrial complex I and rewiring of glutathione metabolism in renal oncocytoma. Proc Natl Acad Sci USA 115: E6283–E6290 29915083 10.1073/pnas.1711888115 PMC6142220

[embr202153054-bib-0019] Gorelick AN , Kim M , Chatila WK , La K , Hakimi AA , Berger MF , Taylor BS , Gammage PA , Reznik E (2021) Respiratory complex and tissue lineage drive recurrent mutations in tumour mtDNA. Nat Metab 3: 558–570 33833465 10.1038/s42255-021-00378-8 PMC9304985

[embr202153054-bib-0020] Guenzler‐Pukall V , Neff TB , Wang Q , Arend MP , Flippin LA , Melekhov A (2003) Stabilization of Hypoxia‐Inducible Factor (HIF) alpha. WO2003049686A2, CAplus AN 2003:472343

[embr202153054-bib-0021] Gustafsson CM , Falkenberg M , Larsson NG (2016) Maintenance and expression of mammalian mitochondrial DNA. Annu Rev Biochem 85: 133–160 27023847 10.1146/annurev-biochem-060815-014402

[embr202153054-bib-0022] Hanahan D , Weinberg RA (2011) Hallmarks of cancer: the next generation. Cell 144: 646–674 21376230 10.1016/j.cell.2011.02.013

[embr202153054-bib-0023] Heitman J , Movva NR , Hall MN (1991) Targets for cell cycle arrest by the immunosuppressant rapamycin in yeast. Science 253: 905–909 1715094 10.1126/science.1715094

[embr202153054-bib-0024] Hensley C , Faubert B , Yuan Q , Lev‐Cohain N , Jin E , Kim J , Jiang L , Ko B , Skelton R , Loudat L *et al* (2016) Metabolic heterogeneity in human lung tumors. Cell 164: 681–694 26853473 10.1016/j.cell.2015.12.034 PMC4752889

[embr202153054-bib-0025] Hillen HS , Temiakov D , Cramer P (2018) Structural basis of mitochondrial transcription. Nat Struct Mol Biol 25: 754–765 30190598 10.1038/s41594-018-0122-9 PMC6583890

[embr202153054-bib-0026] Hu Y , Flockhart I , Vinayagam A , Bergwitz C , Berger B , Perrimon N , Mohr SE (2011) An integrative approach to ortholog prediction for disease‐focused and other functional studies. BMC Bioinformatics 12: 357 21880147 10.1186/1471-2105-12-357 PMC3179972

[embr202153054-bib-0027] Hung C‐M , Garcia‐Haro L , Sparks CA , Guertin DA (2012) mTOR‐dependent cell survival mechanisms. Cold Spring Harb Perspect Biol 4: a008771 23124837 10.1101/cshperspect.a008771 PMC3504431

[embr202153054-bib-0028] Jain IH , Zazzeron L , Goli R , Alexa K , Schatzman‐Bone S , Dhillon H , Goldberger O , Peng J , Shalem O , Sanjana NE *et al* (2016) Hypoxia as a therapy for mitochondrial disease‐ supplementary. Science 352: 54–61 26917594 10.1126/science.aad9642 PMC4860742

[embr202153054-bib-0029] Jain IH , Zazzeron L , Goldberger O , Marutani E , Wojtkiewicz GR , Ast T , Wang H , Schleifer G , Stepanova A , Brepoels K *et al* (2019) Leigh syndrome mouse model can be rescued by interventions that normalize brain hyperoxia, but not HIF activation. Cell Metab 30: 824–832.e3 31402314 10.1016/j.cmet.2019.07.006 PMC6903907

[embr202153054-bib-0030] Jassal B , Matthews L , Viteri G , Gong C , Lorente P , Fabregat A , Sidiropoulos K , Cook J , Gillespie M , Haw R *et al* (2019) The reactome pathway knowledgebase. Nucleic Acids Res 48: D498–D503 10.1093/nar/gkz1031 PMC7145712 31691815

[embr202153054-bib-0031] Jia X , Gu Z , Chen W , Jiao J (2016) Tigecycline targets nonsmall cell lung cancer through inhibition of mitochondrial function. Fundam Clin Pharmacol 30: 297–306 27009695 10.1111/fcp.12199

[embr202153054-bib-0032] Jiang M , Kauppila TES , Motori E , Li X , Atanassov I , Folz‐Donahue K , Bonekamp NA , Albarran‐Gutierrez S , Stewart JB , Larsson N‐G (2017) Increased total mtDNA copy number cures male infertility despite unaltered mtDNA mutation load. Cell Metab 26: 429–436.e4 28768180 10.1016/j.cmet.2017.07.003

[embr202153054-bib-0033] Joharapurkar AA , Pandya VB , Patel VJ , Desai RC , Jain MR (2018) Prolyl hydroxylase inhibitors: a breakthrough in the therapy of anemia associated with chronic diseases. J Med Chem 61: 6964–6982 29712435 10.1021/acs.jmedchem.7b01686

[embr202153054-bib-0034] Ju YS , Alexandrov LB , Gerstung M , Martincorena I , Nik‐Zainal S , Ramakrishna M , Davies HR , Papaemmanuil E , Gundem G , Shlien A *et al* (2014) Origins and functional consequences of somatic mitochondrial DNA mutations in human cancer. Elife 3: e02935 10.7554/eLife.02935 PMC4371858 25271376

[embr202153054-bib-0035] Kaelin Jr WG (2008) The von Hippel‐Lindau tumour suppressor protein: O2 sensing and cancer. Nat Rev Cancer 8: 865–873 18923434 10.1038/nrc2502

[embr202153054-bib-0036] Katigbak A , Cencic R , Robert F , Sénécha P , Scuoppo C , Pelletier J (2016) A CRISPR/Cas9 functional screen identifies rare tumor suppressors. Sci Rep 6: 38968 27982060 10.1038/srep38968 PMC5159885

[embr202153054-bib-0037] Kauppila J , Baines H , Bratic A , Simard M‐L , Freyer C , Mourier A , Stamp C , Filograna R , Larsson N‐G , Greaves L *et al* (2016) A phenotype‐driven approach to generate mouse models with pathogenic mtDNA mutations causing mitochondrial disease. Cell Rep 16: 2980–2990 27626666 10.1016/j.celrep.2016.08.037 PMC5039181

[embr202153054-bib-0038] Kauppila TES , Kauppila JHK , Larsson N‐G (2017) Mammalian mitochondria and aging: an update. Cell Met 25: 57–71 10.1016/j.cmet.2016.09.017 28094012

[embr202153054-bib-0039] Khan NA , Nikkanen J , Yatsuga S , Jackson C , Wang L , Pradhan S , Kivelä R , Pessia A , Velagapudi V , Suomalainen A (2017) mTORC1 regulates mitochondrial integrated stress response and mitochondrial myopathy progression. Cell Metab 26: 419–428.e5 28768179 10.1016/j.cmet.2017.07.007

[embr202153054-bib-0040] Kuntz EM , Baquero P , Michie AM , Dunn K , Tardito S , Holyoake TL , Helgason GV , Gottlieb E (2017) Targeting mitochondrial oxidative phosphorylation eradicates therapy‐resistant chronic myeloid leukemia stem cells. Nat Med 23: 1234–1240 28920959 10.1038/nm.4399 PMC5657469

[embr202153054-bib-0041] Larsson N‐G , Clayton DA (1995) Molecular genetic aspects of human mitochondrial disorders. Annu Rev Genet 29: 151–178 8825472 10.1146/annurev.ge.29.120195.001055

[embr202153054-bib-0042] Lee J , Yesilkanal AE , Wynne JP , Frankenberger C , Liu J , Yan J , Elbaz M , Rabe DC , Rustandy FD , Tiwari P *et al* (2019) Effective breast cancer combination therapy targeting BACH1 and mitochondrial metabolism. Nature 568: 254–258 30842661 10.1038/s41586-019-1005-x PMC6698916

[embr202153054-bib-0043] Maxwell PH , Wiesener MS , Chang G‐W , Clifford SC , Vaux EC , Cockman ME , Wykoff CC , Pugh CW , Maher ER , Ratcliffe PJ (1999) The tumour suppressor protein VHL targets hypoxia‐inducible factors for oxygen‐dependent proteolysis. Nature 399: 271–275 10353251 10.1038/20459

[embr202153054-bib-0044] McDermott M , Eustace AJ , Busschots S , Breen L , Crown J , Clynes M , O’Donovan N , Stordal B (2014) In vitro development of chemotherapy and targeted therapy drug‐resistant cancer cell lines: a practical guide with case studies. Front Oncol 4: 40 24639951 10.3389/fonc.2014.00040 PMC3944788

[embr202153054-bib-0045] McKee EE , Ferguson M , Bentley AT , Marks TA (2006) Inhibition of mammalian mitochondrial protein synthesis by oxazolidinones. Antimicrob Agents Chemother 50: 2042–2049 16723564 10.1128/AAC.01411-05 PMC1479116

[embr202153054-bib-0046] Minchinton AI , Tannock IF (2006) Drug penetration in solid tumours. Nat Rev Cancer 6: 583–592 16862189 10.1038/nrc1893

[embr202153054-bib-0047] Molina JR , Sun Y , Protopopova M , Gera S , Bandi M , Bristow C , McAfoos T , Morlacchi P , Ackroyd J , Agip A‐NA *et al* (2018) An inhibitor of oxidative phosphorylation exploits cancer vulnerability. Nat Med 24: 1036–1046 29892070 10.1038/s41591-018-0052-4

[embr202153054-bib-0048] Papandreou I , Cairns RA , Fontana L , Lim AL , Denko NC (2006) HIF‐1 mediates adaptation to hypoxia by actively downregulating mitochondrial oxygen consumption. Cell Metab 3: 187–197 16517406 10.1016/j.cmet.2006.01.012

[embr202153054-bib-0049] Perez‐Riverol Y , Csordas A , Bai J , Bernal‐Llinares M , Hewapathirana S , Kundu DJ , Inuganti A , Griss J , Mayer G , Eisenacher M *et al* (2019) The PRIDE database and related tools and resources in 2019: improving support for quantification data. Nucleic Acids Res 47: D442 30395289 10.1093/nar/gky1106 PMC6323896

[embr202153054-bib-0050] Podsypanina K , Lee RT , Politis C , Hennessy I , Crane A , Puc J , Neshat M , Wang H , Yang L , Gibbons J *et al* (2001) An inhibitor of mTOR reduces neoplasia and normalizes p70/S6 kinase activity in Pten+/‐ mice. Proc Natl Acad Sci USA 98: 10320–10325 11504907 10.1073/pnas.171060098 PMC56959

[embr202153054-bib-0051] Rabinowitz MH (2013) Inhibition of hypoxia‐inducible factor prolyl hydroxylase domain oxygen sensors: tricking the body into mounting orchestrated survival and repair responses. J Med Chem 56: 9369–9402 23977883 10.1021/jm400386j

[embr202153054-bib-0052] Reed GA , Schiller GJ , Kambhampati S , Tallman MS , Douer D , Minden MD , Yee KW , Gupta V , Brandwein J , Jitkova Y *et al* (2016) A Phase 1 study of intravenous infusions of tigecycline in patients with acute myeloid leukemia. Cancer Med 5: 3031–3040 27734609 10.1002/cam4.845 PMC5119957

[embr202153054-bib-0053] Reznik ED , Miller ML , Şenbabaoğlu Y , Riaz N , Sarungbam J , Tickoo SK , Al‐Ahmadie HA , Lee W , Seshan VE , Hakimi AA *et al* (2016) Mitochondrial DNA copy number variation across human cancers. Elife 5: 1–20 10.7554/eLife.10769 PMC4775221 26901439

[embr202153054-bib-0054] Rini BI (2008) Temsirolimus, an inhibitor of mammalian target of rapamycin. Clin Cancer Res 14: 1286–1290 18316545 10.1158/1078-0432.CCR-07-4719

[embr202153054-bib-0055] Sayed S , Paszkowski‐Rogacz M , Schmitt LT , Buchholz F (2019) CRISPR/Cas9 as a tool to dissect cancer mutations. Methods 165: 36–48 10.1016/j.ymeth.2019.05.007 31078796

[embr202153054-bib-0056] Schmierer B , Botla SK , Zhang J , Turunen M , Kivioja T , Taipale J (2017) CRISPR/Cas9 screening using unique molecular identifiers. Mol Syst Biol 13: 945 28993443 10.15252/msb.20177834 PMC5658704

[embr202153054-bib-0057] Sehgal SN , Baker H , Vézina C (1975) Rapamycin (AY‐22,989), a new antifungal antibiotic. II. Fermentation, isolation and characterization. J Antibiot 28: 727–732 10.7164/antibiotics.28.727 1102509

[embr202153054-bib-0058] Shalem O , Sanjana NE , Hartenian E , Shi X , Scott DA , Mikkelsen TS , Heckl D , Ebert BL , Root DE , Doench JG *et al* (2014) Genome‐scale CRISPR‐Cas9 knockout screening in human cells. Science 343: 84–87 24336571 10.1126/science.1247005 PMC4089965

[embr202153054-bib-0059] Sharma S , Petsalaki E (2018) Application of CRISPR‐Cas9 based genome‐wide screening approaches to study cellular signalling mechanisms. Int J Mol Sci 19: 933 10.3390/ijms19040933 PMC5979383 29561791

[embr202153054-bib-0060] Shi Y , Lim SK , Liang Q , Iyer SV , Wang H‐Y , Wang Z , Xie X , Sun D , Chen Y‐J , Tabar V *et al* (2019) Gboxin is an oxidative phosphorylation inhibitor that targets glioblastoma HHS public access. Nature 567: 341–346 30842654 10.1038/s41586-019-0993-x PMC6655586

[embr202153054-bib-0061] Škrtić M , Sriskanthadevan S , Jhas B , Gebbia M , Wang X , Wang Z , Hurren R , Jitkova Y , Gronda M , Maclean N *et al* (2011) Inhibition of mitochondrial translation as a therapeutic strategy for human acute myeloid leukemia. Cancer Cell 20: 674–688 22094260 10.1016/j.ccr.2011.10.015 PMC3221282

[embr202153054-bib-0062] Sterky FH , Hoffman AF , Milenkovic D , Bao B , Paganelli A , Edgar D , Wibom R , Lupica CR , Olson L , Larsson N‐G (2012) Altered dopamine metabolism and increased vulnerability to MPTP in mice with partial deficiency of mitochondrial complex I in dopamine neurons. Hum Mol Genet 21: 1078–1089 22090423 10.1093/hmg/ddr537 PMC3277308

[embr202153054-bib-0063] Stewart JB , Alaei‐Mahabadi B , Sabarinathan R , Samuelsson T , Gorodkin J , Gustafsson CM , Larsson E (2015) Simultaneous DNA and RNA mapping of somatic mitochondrial mutations across diverse human cancers. PLoS Genet 11: 1–15 10.1371/journal.pgen.1005333 PMC4488357 26125550

[embr202153054-bib-0064] Stewart JB , Chinnery PF (2015) The dynamics of mitochondrial DNA heteroplasmy: implications for human health and disease. Nat Rev Genet 16: 530–542 26281784 10.1038/nrg3966

[embr202153054-bib-0065] Sun S‐Y (2013) mTOR kinase inhibitors as potential cancer therapeutic drugs. Cancer Lett 340: 1–8 23792225 10.1016/j.canlet.2013.06.017 PMC3779533

[embr202153054-bib-0066] Swanton C (2012) Intratumor heterogeneity: evolution through space and time. Cancer Res 72: 4875–4882 23002210 10.1158/0008-5472.CAN-12-2217 PMC3712191

[embr202153054-bib-0067] Szakács G , Paterson JK , Ludwig JA , Booth‐Genthe C , Gottesman MM (2006) Targeting multidrug resistance in cancer. Nat Rev Drug Discov 5: 219–234 16518375 10.1038/nrd1984

[embr202153054-bib-0068] Thimmaiah KN , Easton JB , Houghton PJ (2010) Protection from rapamycin‐induced apoptosis by insulin‐like growth factor‐I is partially dependent on protein kinase C signaling. Cancer Res 70: 2000–2009 20179209 10.1158/0008-5472.CAN-09-3693 PMC3003869

[embr202153054-bib-0069] Valle S , Martin‐Hijano L , Alcalá S , Alonso‐Nocelo M , Sainz Jr B (2018) The ever‐evolving concept of the cancer stem cell in pancreatic cancer. Cancers 10: 33 10.3390/cancers10020033 PMC5836065 29373514

[embr202153054-bib-0070] Vander Heiden MG , Cantley LC , Thompson CB (2009) Understanding the Warburg effect: the metabolic requirements of cell proliferation. Science 324: 1029–1033 19460998 10.1126/science.1160809 PMC2849637

[embr202153054-bib-0071] Vasan K , Werner M , Chandel NS (2020) Mitochondrial metabolism as a target for cancer therapy. Cell Metab 32: 341–352 32668195 10.1016/j.cmet.2020.06.019 PMC7483781

[embr202153054-bib-0072] Viale A , Pettazzoni P , Lyssiotis CA , Ying H , Sánchez N , Marchesini M , Carugo A , Green T , Seth S , Giuliani V *et al* (2014) Oncogene ablation‐resistant pancreatic cancer cells depend on mitochondrial function. Nature 514: 628–632 25119024 10.1038/nature13611 PMC4376130

[embr202153054-bib-0073] Villar VH , Nguyen TL , Delcroix V , Terés S , Bouchecareilh M , Salin B , Bodineau C , Vacher P , Priault M , Soubeyran P *et al* (2017) mTORC1 inhibition in cancer cells protects from glutaminolysis‐mediated apoptosis during nutrient limitation. Nat Commun 8: 14124 28112156 10.1038/ncomms14124 PMC5264013

[embr202153054-bib-0074] Vögtle F‐N , Burkhart JM , Gonczarowska‐Jorge H , Kücükköse C , Taskin AA , Kopczynski D , Ahrends R , Mossmann D , Sickmann A , Zahedi RP *et al* (2017) Landscape of submitochondrial protein distribution. Nat Commun 8: 290 28819139 10.1038/s41467-017-00359-0 PMC5561175

[embr202153054-bib-0075] Wang GL , Semenza GL (1993) General involvement of hypoxia‐inducible factor 1 in transcriptional response to hypoxia. Proc Natl Acad Sci USA 90: 4304–4308 8387214 10.1073/pnas.90.9.4304 PMC46495

[embr202153054-bib-0076] Woodward GE , Hudson MT (1954) The effect of 2‐desoxy‐d‐glucose on glycolysis and respiration of tumor and normal tissues. Cancer Res 14: 599–605 13199805

[embr202153054-bib-0077] Yang J , Zhou R , Ma Z (2019) Autophagy and energy metabolism. Adv Exp Med Biol 1206: 329–357 31776993 10.1007/978-981-15-0602-4_16

[embr202153054-bib-0078] Yuan Y , Ju YS , Kim Y , Li J , Wang Y , Yoon CJ , Yang Y , Martincorena I , Creighton CJ , Weinstein JN *et al* (2020) Comprehensive molecular characterization of mitochondrial genomes in human cancers. Nat Genet 52: 342–352 32024997 10.1038/s41588-019-0557-x PMC7058535

[embr202153054-bib-0079] Zhang H , Gao P , Fukuda R , Kumar G , Krishnamachary B , Zeller KI , Dang CV , Semenza GL (2007) HIF‐1 inhibits mitochondrial biogenesis and cellular respiration in VHL‐deficient renal cell carcinoma by repression of C‐MYC activity. Cancer Cell 11: 407–420 17482131 10.1016/j.ccr.2007.04.001

[embr202153054-bib-0080] Zheng X , Boyer L , Jin M , Kim Y , Fan W , Bardy C , Berggren T , Evans RM , Gage FH , Hunter T (2016) Alleviation of neuronal energy deficiency by mTOR inhibition as a treatment for mitochondria‐related neurodegeneration. Elife 5: e13378 27008180 10.7554/eLife.13378 PMC4846388

[embr202153054-bib-0081] Zhou T , Sang Y‐H , Cai S , Xu C , Shi M (2021) The requirement of mitochondrial RNA polymerase for non‐small cell lung cancer cell growth. Cell Death Dis 128: 1–12 10.1038/s41419-021-04039-2 PMC8322058 34326320

