# Supplementary material for: Metabolic resistance to the inhibition of mitochondrial transcription revealed by CRISPR‐Cas9 screen
Source: EMBO Rep. 2021 Nov 15;23(1):e53054. doi: 10.15252/embr.202153054 (PMC8728608; doi:10.15252/embr.202153054)
Supplement: Supplementary file 1 — Expanded View Figures PDF [file EMBR-23-e53054-s004.pdf]

Expanded View Figures

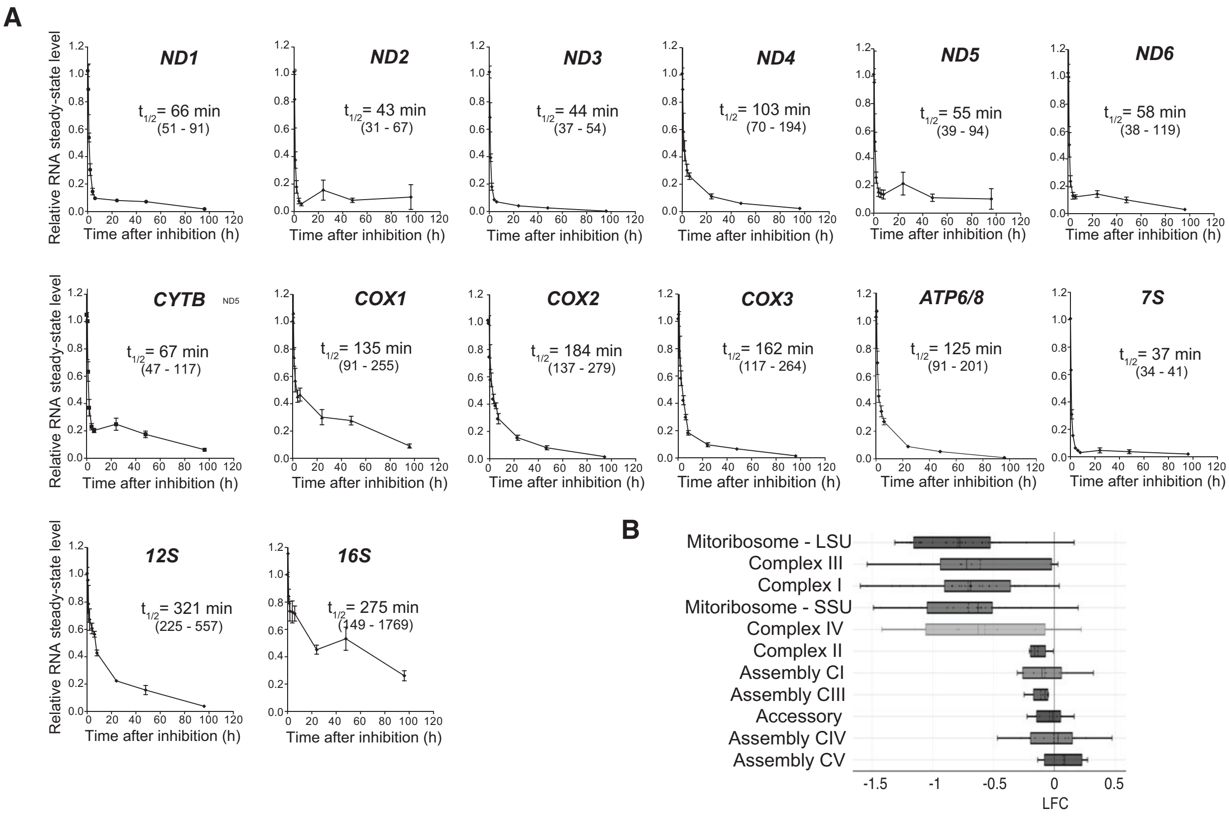

Figure EV1. IMT1 treatment causes a fast drop in mitochondrial transcript levels.

A Quantitative real-time polymerase chain reaction (qRT–PCR) assessment of mitochondrial transcript levels followed over time in HeLa cells (0–96 h). Half-life was assessed in semi-logarithmic plot using non-linear regression curve fit. The estimated transcript half-lives are shown in minutes, the 95% confidence interval is indicated in brackets. Data are plotted as mean ± SEM of n = 4 independent experiments.

B Box plots showing changes in protein levels among submitochondrial compartments, according to Vögtle *et al*, 2017. Data are expressed as log_2-fold change (LFC) of controls (dimethyl sulfoxide (DMSO)-treated RKO cells) from n = 3 independent experiments. Median (solid line) and mean (dashed line), upper and lower quartile, and 1.5× interquartile range (whiskers).

© 2021 The Authors

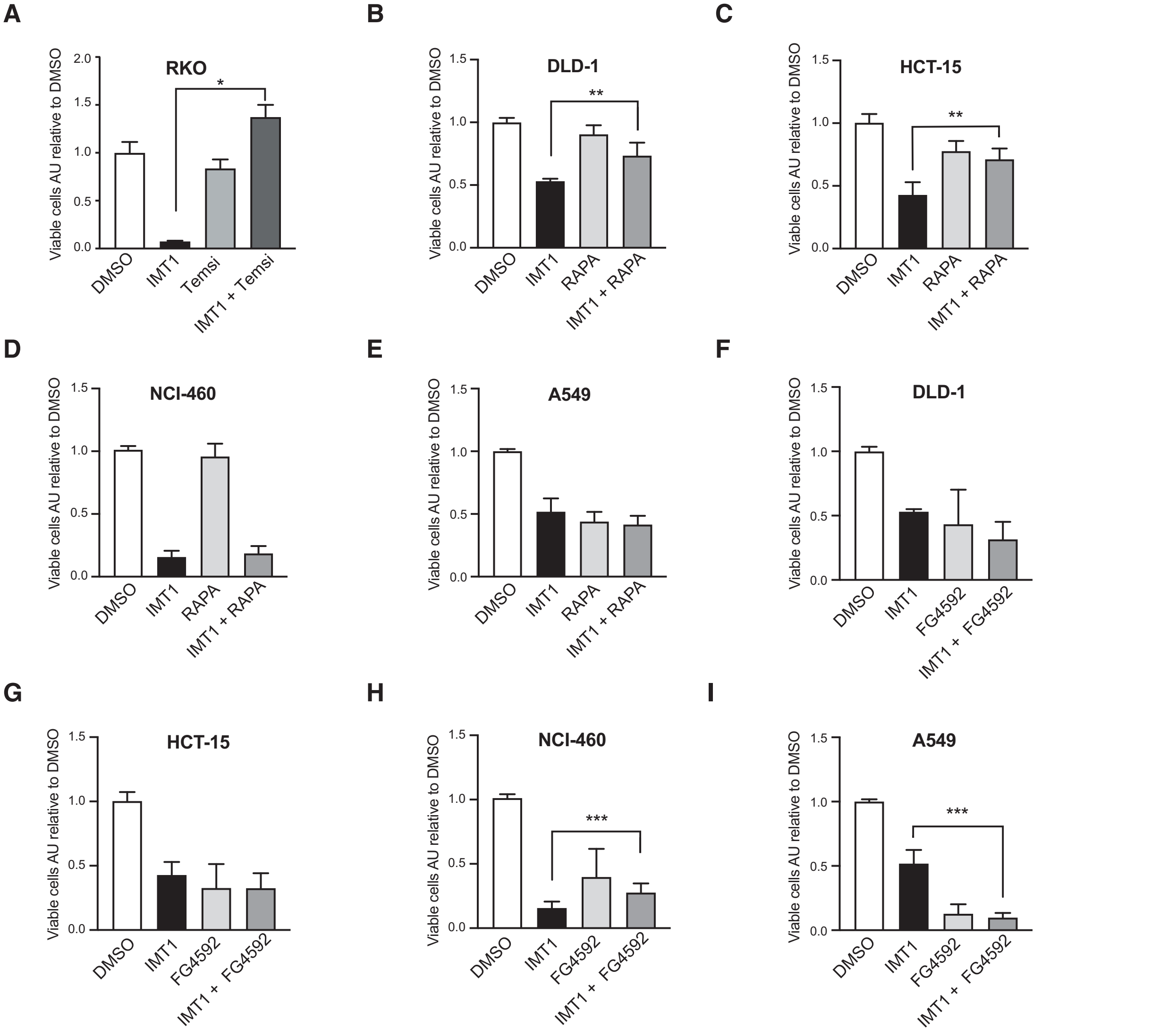

Figure EV2. Rapamycin and FG4592 increase tolerance to IMT1 treatment in several cancer cell lines.

A Effect of temsirolimus alone or in combination with IMT1 on RKO cell viability. Mean ± SD of $n = 3$ independent experiments, one-way ANOVA, IMT1 versus IMT1+ Temsirolimus: *$P = 0.0278$.

B–E Cell viability assessment of a panel of IMT1-sensitive cancer cell lines treated with IMT1 alone and in combination with rapamycin (DLD-1 (B), HCT-15 (C), NCI-460 (D), and A549 (E)). Data are expressed as mean values ± SD of $n = 3$ independent experiments, each including four technical intra-plate replicates. Statistical significance was calculated with one-way ANOVA test. IMT1 versus IMT1 + RAPA, DLD-1: **$P = 0.0057$; HCT-15: **$P = 0.046$; NCI-460 and A549 non-significant.

F–I Cell viability assessment of a panel of IMT1-sensitive cancer cell lines treated with IMT1 alone and in combination with FG4592 (DLD-1 (F), HCT-15 (G), NCI-460 (H), and A549 (I)). Data are expressed as mean values ± SD of $n = 3$ independent experiments, each including four technical intra-plate replicates, statistical significance was calculated with one-way ANOVA test. IMT1 versus IMT1 + FG4592, NCI-460: ***$P = 0.0003$; A549: ***$P < 0.0001$; DLD-1 and HCT-15: non-significant.

 © 2021 The Authors

Figure EV3. Increased mitochondrial biogenesis and changes in autophagy do not explain IMT1 resistance induced by rapamycin and FG4592.

A, B Quantitative real-time polymerase chain reaction (qRT–PCR) assessment of mitochondrial DNA (mtDNA) (A) and transcripts (B) levels in RKO cells treated with dimethyl sulfoxide (DMSO), IMT1, rapamycin, and FG4592 for 3 days. Data are relative to DMSO-treated controls and expressed as mean ± SEM of $n = 4$ independent experiments.

C Relative peroxisome proliferator-activated receptor gamma coactivator 1 alpha (PGC1α) messenger RNA (mRNA) levels in RKO cells treated as described in A; mean ± SEM of $n = 3$ independent experiments.

D–G Quantitative real-time polymerase chain reaction (qRT–PCR) assessment of mitochondrial DNA (mtDNA) (D, F) and transcripts (E, G) levels in MiaPaCa-2 (D, E) and HeLa (F, G) cells treated with dimethyl sulfoxide (DMSO), IMT1, rapamycin, and FG4592 for 3 days. Data are relative to DMSO-treated controls and expressed as mean ± SEM of $n = 2$ independent experiments.

H Representative western blot analyses of microtubule-associated proteins 1A/1B light chain 3B (LC3BII) accumulation after blocking the autophagosome acidification with NH_4Cl for 3 h in RKO cells in the presence of dimethyl sulfoxide (DMSO), IMT1, rapamycin, or FG4592.

I Densitometric quantification of microtubule-associated proteins 1A/1B light chain 3B (LC3BII) levels plotted as mean values ± SD from $n = 4$ independent experiments.

© 2021 The Authors

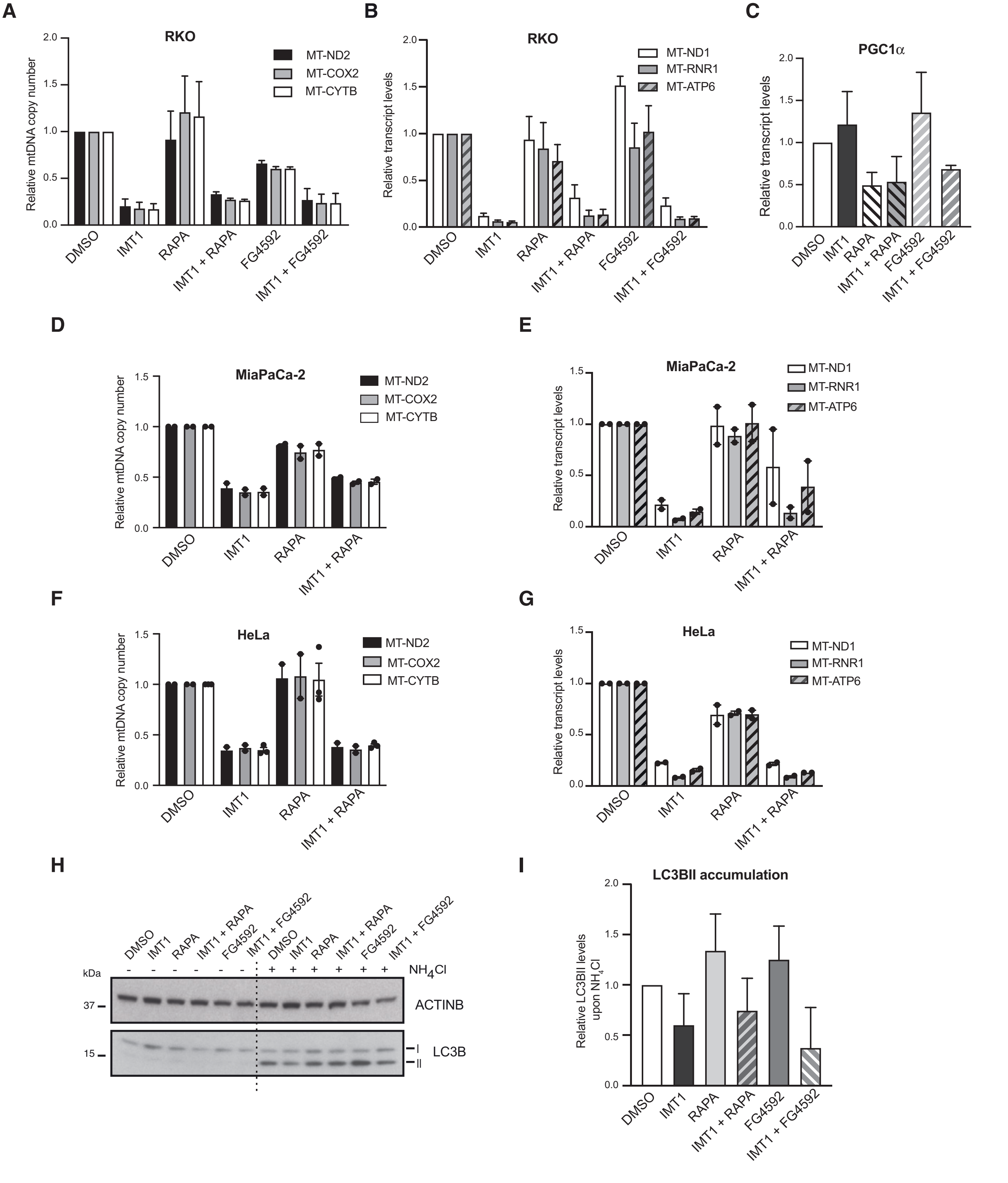

Figure EV3.

 © 2021 The Authors

Figure EV4. The inhibition of mitochondrial translation and decrease in mitochondrial DNA (mtDNA) copy number represent a tool to target IMT1 resistance.

A Dose–response curves of monolayer cultures of RKO cells at increasing chloramphenicol (CAP) concentrations for 1 week, viability was determined as the ratio of dimethyl sulfoxide (DMSO)-treated controls. Data are expressed as mean values ± SD of *n* = 3 independent experiments, each including three technical intra-plate replicates.

B Representative western blot assessments of mitochondrial (mt)-encoded cytochrome c oxidase subunit 2 (COX2) protein levels at increasing chloramphenicol (CAP) concentrations, compared to ATP synthase F1 subunit alpha (ATP5A) and glyceraldehyde 3-phosphate dehydrogenase (GAPDH) for loading reference.

C Viability assessment of RKO cells treated for 1 week with either dimethyl sulfoxide (DMSO), IMT1 (1 μM), chloramphenicol (CAP) (1 μg/ml) alone or in combination with IMT1. Data are expressed as mean values ± SD of *n* = 3 independent experiments, each including four technical intra-plate replicates. Statistical significance was calculated with one-way ANOVA test. DMSO versus IMT1: **P* = 0.0159, DMSO versus IMT1+CAP: **P* = 0.0264, DMSO versus CAP and IMT1 versus IMT1+CAP non-significant.

D Cell viability assessment of human primary fibroblasts from two healthy individuals treated for 1 week with either dimethyl sulfoxide (DMSO), IMT1 (1 μM), chloramphenicol (CAP) (1 μg/ml) alone or in combination with IMT1. Data are the mean values ± SD of *n* = 3 independent experiments. One-way ANOVA showed no significant difference.

E Dose–response curve of spheroidal growth of parental RKO cells at increasing chloramphenicol (CAP) concentrations for 2 weeks expressed as spheroid areas (mm^2). Data represent mean values ± SD of *n* = 3 independent experiments. Representative images of the effect of serial CAP concentrations on spheroidal growth in parental RKOs are reported below the graph; scale bar: 1 mm.

F Dose–response spheroidal growth in RKO cells at increasing IMT1 and chloramphenicol (CAP) concentrations; scale bar: 1 mm.

G, H Log_2-fold changes (LFCs) in viable cell counts in a panel of five IMT1-resistant cancer cell lines in the presence of IMT1 + 100 μg/ml of chloramphenicol (CAP) (G) and IMT1 + 1 mM 2-deoxy-D-glucose (2DG) (H). Data represent the mean values of *n* = 3 ±SD independent experiments; paired t-test of IMT1 versus IMT1+CAP (100 μg/ml), resistant RKO: ***P* = 0.0068, PANC-1: ***P* = 0.0098, Capan-2: non-significant and HCT-29: **P* = 0.0431, Calu-6: non-significant. 2DG + IMT1 versus IMT1: non-significant.

I Western blot analyses of mitochondrial transcription factor A (TFAM) protein steady-state levels over time after knockdown with two independent small interfering RNAs (siRNAs) against TFAM (TFAM #1 and #2) or controls (Control #1, #2) in IMT1-resistant RKO cells.

J Changes in mitochondrial DNA (mtDNA) levels after mitochondrial transcription factor A (TFAM) downregulation performed as in panel I.

© 2021 The Authors

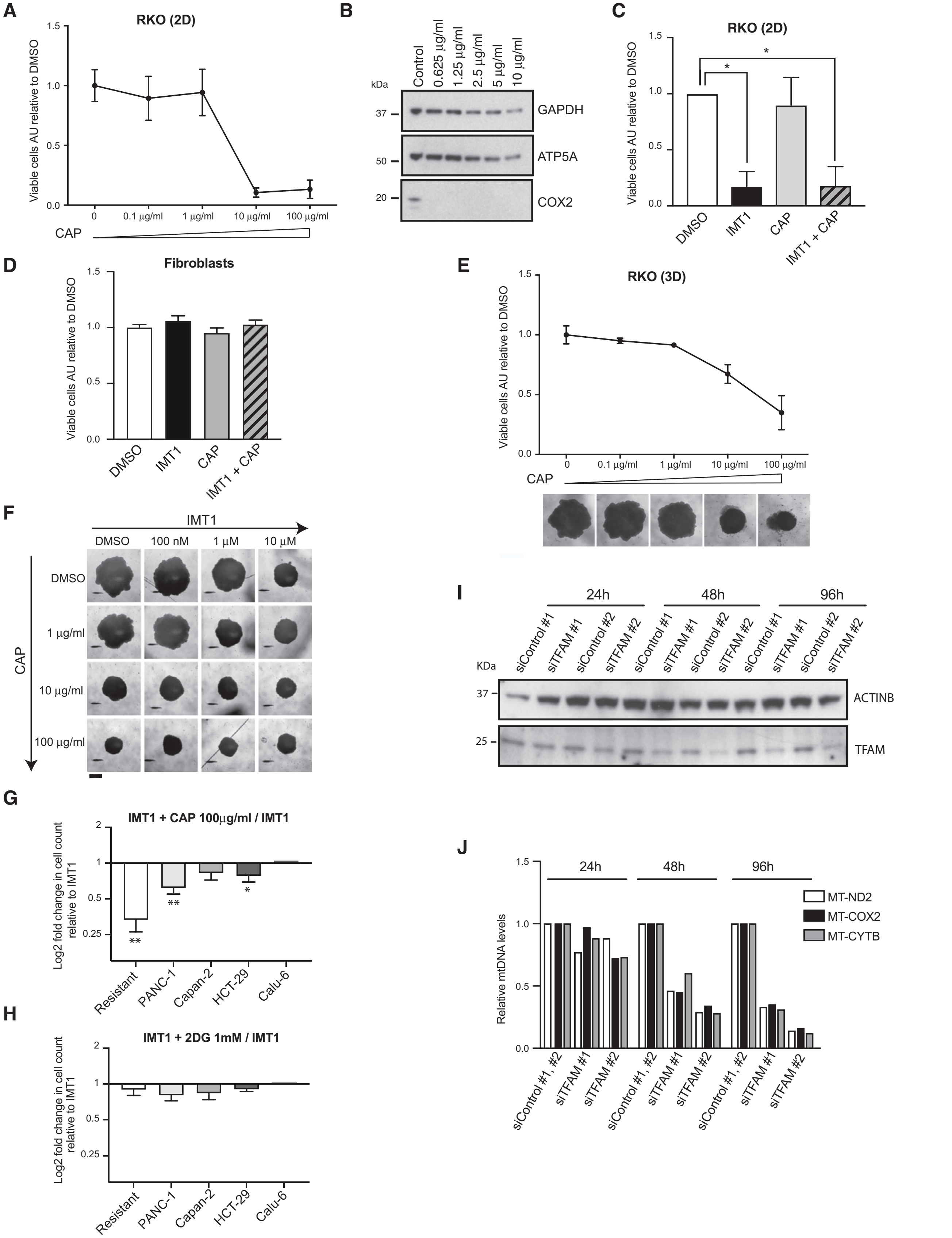

Figure EV4.

© 2021 The Authors
