# Supplementary material for: Metabolic resistance to the inhibition of mitochondrial transcription revealed by CRISPR‐Cas9 screen
Source: EMBO Rep. 2021 Nov 15;23(1):e53054. doi: 10.15252/embr.202153054 (PMC8728608; doi:10.15252/embr.202153054)
Supplement: Supplementary file 5 [file EMBR-23-e53054-s001.pdf]

EMBO reports

Metabolic resistance to the inhibition of mitochondrial transcription revealed by CRISPR-Cas9 screen

Mara Mennuni, Roberta Filograna, Andrea Felser, Nina Bonekamp, Patrick Giavalisco, Oleksandr Lytovchenko, and Nils-Göran Larsson
DOI: 10.15252/embr.202153054

Corresponding author(s): Nils-Göran Larsson (nils-goran.larsson@ki.se)

Review Timeline:

Submission Date:	14th Apr 21
Editorial Decision:	10th May 21
Revision Received:	8th Sep 21
Editorial Decision:	8th Oct 21
Revision Received:	12th Oct 21
Accepted:	19th Oct 21

Editor: Deniz Senyilmaz Tiebe

Transaction Report:

(Note: With the exception of the correction of typographical or spelling errors that could be a source of ambiguity, letters and reports are not edited. Depending on transfer agreements, referee reports obtained elsewhere may or may not be included in this compilation. Referee reports are anonymous unless the Referee chooses to sign their reports.)

1st Editorial Decision 10th May 2021

Dear Dr. Larsson,

Thank you for the submission of your research manuscript to our journal, which was now seen by three referees, whose reports are copied below.

We concur with the referees that the proposed modulators of IMT1 resistance in cancer cells is in principle very interesting. However, referees raise largely overlapping concerns that need to be addressed to consider publication here. In particular, referees find that some mechanistic insight into how VHL and mTORC1 inhibition affects IMT1 resistance is required and they propose some possible mechanisms that could be at play to be tested. We agree with the referees that this would strengthen the manuscript significantly.

Given these positive recommendations, we would like to invite you to revise your manuscript with the understanding that the referee concerns (as in their reports) must be fully addressed and their suggestions taken on board. Please address all referee concerns in a complete point-by-point response. Acceptance of the manuscript will depend on a positive outcome of a second round of review. It is EMBO reports policy to allow a single round of revision only and acceptance or rejection of the manuscript will therefore depend on the completeness of your responses included in the next, final version of the manuscript.

We generally allow three months as standard revision time. As a matter of policy, competing manuscripts published during this period will not negatively impact on our assessment of the conceptual advance presented by your study. However, we request that you contact the editor as soon as possible upon publication of any related work, to discuss how to proceed. Should you foresee a problem in meeting this three-month deadline, please let us know in advance and we may be able to grant an extension.

*** Temporary update to EMBO Press scooping protection policy:
We are aware that many laboratories cannot function at full efficiency during the current COVID-19/SARS-CoV-2 pandemic and have therefore extended our 'scooping protection policy' to cover the period required for a full revision to address the experimental issues highlighted in the editorial decision letter. Please contact the scientific editor handling your manuscript to discuss a revision plan should you need additional time, and also if you see a paper with related content published elsewhere.***

IMPORTANT NOTE: we perform an initial quality control of all revised manuscripts before re-review. Your manuscript will FAIL this control and the handling will be DELAYED if the following APPLIES:
1. A data availability section providing access to data deposited in public databases is missing (where applicable).
2. Your manuscript contains statistics and error bars based on n=2. Please use scatter plots in these cases.

You can submit the revision either as a Scientific Report or as a Research Article. For Scientific Reports, the revised manuscript can contain up to 5 main figures and 5 Expanded View figures. If the revision leads to a manuscript with more than 5 main figures it will be published as a Research Article. In this case the Results and Discussion section should be separate. If a Scientific Report is submitted, these sections have to be combined. This will help to shorten the manuscript text by eliminating some redundancy that is inevitable when discussing the same experiments twice. In either case, all materials and methods should be included in the main manuscript file

Supplementary/additional data: The Expanded View format, which will be displayed in the main HTML of the paper in a collapsible format, has replaced the Supplementary information. You can submit up to 5 images as Expanded View. Please follow the nomenclature Figure EV1, Figure EV2 etc. The figure legend for these should be included in the main manuscript document file in a section called Expanded View Figure Legends after the main Figure Legends section. Additional Supplementary material should be supplied as a single pdf labeled Appendix. The Appendix includes a table of content on the first page with page numbers, all figures and their legends. Please follow the nomenclature Appendix Figure Sx throughout the text and also label the figures according to this nomenclature. For more details please refer to our guide to authors.

Please note that for all articles published beginning 1 July 2020, the EMBO Reports reference style will change to the Harvard style for all article types. Details and examples are provided at
https://www.embopress.org/page/journal/14693178/authorguide#referencesformat

When submitting your revised manuscript, please carefully review the instructions that follow below. Failure to include requested items will delay the evaluation of your revision.

1) a .docx formatted version of the manuscript text (including legends for main figures, EV figures and tables). Please make sure that the changes are highlighted to be clearly visible.

2) individual production quality figure files as .eps, .tif, .jpg (one file per figure).

3) a .docx formatted letter INCLUDING the reviewers' reports and your detailed point-by-point responses to their comments. As

part of the EMBO Press transparent editorial process, the point-by-point response is part of the Review Process File (RPF), which will be published alongside your paper. For more details on our Transparent Editorial Process, please visit our website: https://www.embopress.org/page/journal/14693178/authorguide#transparentprocess
You are able to opt out of this by letting the editorial office know (emboreports@embo.org). If you do opt out, the Review Process File link will point to the following statement: "No Review Process File is available with this article, as the authors have chosen not to make the review process public in this case."

4) a complete author checklist, which you can download from our author guidelines (). Please insert information in the checklist that is also reflected in the manuscript. The completed author checklist will also be part of the RPF.

5) Please note that all corresponding authors are required to supply an ORCID ID for their name upon submission of a revised manuscript (). Please find instructions on how to link your ORCID ID to your account in our manuscript tracking system in our Author guidelines ().

6) We replaced Supplementary Information with Expanded View (EV) Figures and Tables that are collapsible/expandable online. A maximum of 5 EV Figures can be typeset. EV Figures should be cited as 'Figure EV1, Figure EV2" etc... in the text and their respective legends should be included in the main text after the legends of regular figures.

- For the figures that you do NOT wish to display as Expanded View figures, they should be bundled together with their legends in a single PDF file called *Appendix*, which should start with a short Table of Content. Appendix figures should be referred to in the main text as: "Appendix Figure S1, Appendix Figure S2" etc. See detailed instructions regarding expanded view here: .

- Additional Tables/Datasets should be labeled and referred to as Table EV1, Dataset EV1, etc. Legends have to be provided in a separate tab in case of .xls files. Alternatively, the legend can be supplied as a separate text file (README) and zipped together with the Table/Dataset file.

7) We would also encourage you to include the source data for figure panels that show essential data.

Numerical data should be provided as individual .xls or .csv files (including a tab describing the data). For blots or microscopy, uncropped images should be submitted (using a zip archive if multiple images need to be supplied for one panel). Additional information on source data and instruction on how to label the files are available .

8) Our journal encourages inclusion of *data citations in the reference list* to directly cite datasets that were re-used and obtained from public databases. Data citations in the article text are distinct from normal bibliographical citations and should directly link to the database records from which the data can be accessed. In the main text, data citations are formatted as follows: "Data ref: Smith et al, 2001" or "Data ref: NCBI Sequence Read Archive PRJNA342805, 2017". In the Reference list, data citations must be labeled with "[DATASET]". A data reference must provide the database name, accession number/identifiers and a resolvable link to the landing page from which the data can be accessed at the end of the reference. Further instructions are available at .

9) Please make sure to include a Data Availability Section before submitting your revision - if it is not applicable, make a statement that no data were deposited in a public database. Primary datasets (and computer code, where appropriate) produced in this study need to be deposited in an appropriate public database (see).

Please remember to provide a reviewer password if the datasets are not yet public.

The accession numbers and database should be listed in a formal "Data Availability " section (placed after Materials & Method) that follows the model below. Please note that the Data Availability Section is restricted to new primary data that are part of this study.

Data availability

The datasets (and computer code) produced in this study are available in the following databases:

- RNA-Seq data: Gene Expression Omnibus GSE46843 (https://www.ncbi.nlm.nih.gov/geo/query/acc.cgi?acc=GSE46843)
- [data type]: [name of the resource] [accession number/identifier/doi] ([URL or identifiers.org/DATABASE:ACCESSION])

*** Note - All links should resolve to a page where the data can be accessed. ***

10) Regarding data quantification, please ensure to specify the name of the statistical test used to generate error bars and P values, the number (n) of independent experiments underlying each data point (not replicate measures of one sample), and the

test used to calculate p-values in each figure legend. Discussion of statistical methodology can be reported in the materials and methods section, but figure legends should contain a basic description of n, P and the test applied.
Please note that error bars and statistical comparisons may only be applied to data obtained from at least three independent biological replicates.
Please also include scale bars in all microscopy images.

We would also welcome the submission of cover suggestions, or motifs to be used by our Graphics Illustrator in designing a cover.

I look forward to seeing a revised version of your manuscript when it is ready. Please let me know if you have questions or comments regarding the revision.

Kind regards,

Deniz

Deniz Senyilmaz Tiebe, PhD
Editor
EMBO Reports

Referee #1:

The manuscript by Mennuni et al. investigates resistance mechanisms to inhibitors of mitochondrial transcription (IMTs). IMTs were previously discovered by the same group to robustly inhibit POLRMT mtDNA gene expression. In their previous studies, the authors found anti-tumor properties of IMTs and in the present manuscript they employed CRISPR-Cas9 screening to search for pathways involved in resistance to IMTs treatment. Authors found VHL and mTORC1 pathways to be involved in IMTs resistance and they showed that IMTs resistance relies on upregulation of mitochondrial gene expression. Overall I find the paper straightforward to read, I like the focused CRISPR results, and points to resistance mechanisms which are important to be cognizant of in the context of future translational studies.

Comments:

-Authors found VHL and mTORC1 pathway inhibition as a source of IMTs resistance. What would be a proposed mechanism of the resistance? Since authors observe increased levels of mitochondrial oxphos proteins by rapamycin treatment is it by decreased mitophagy, increased mitochondrial biogenesis or altered protein stability? Are the mechanisms convergent or distinct between vHL and mTOR? Is it clear why vHL was previously identified in CRISPR screens but mTOR pathway was not? I would love to see the authors develop a bit more the mechanisms here linking VHL or mTOR and mitochondria.

-Introduction lines 55-58: In contrast to the author's note, only in certain tumor types (colorectal, renal, thyroid) have mtDNA mutations shown to be enriched with VAF patterns and signatures of selection compatible with "driver" properties (Gammage and Frezza, BMC, 2019, Gorelick et al., Nat Metab., 2021, Gopal et al., PNAS, 2018). Utility of IMTs for cancer treatment may be restricted to this subset.

-Results lines 88-90: Authors note that one third of previously studied cancer cell lines are sensitive to IMTs. It would be interesting to add a line of comment why these particular cell lines are sensitive while other are not -- can they now go back and explain this on the basis of either the VHL or MTOR pathway?

-Did the authors study if IMT1-resistant cells rely on oxphos vs glycolysis? How does it compare to IMT1-sensitive cells and cells with VHL

Referee #2:

The authors have recently developed small molecule inhibitors of the mitochondrial RNA polymerase (POLMRT)(Bonemkamp et al., 2020). These inhibitors of mitochondrial transcription (IMTs) repress cancer cell proliferation in vitro and delay tumour growth in xenografts of human cancer cells in the mouse. However, from a panel of 89 cancer cell lines, they found that approximately two thirds were resistant to IMTs.

In the present paper, the authors study the mechanisms underlying resistance to their lead compound IMT1 in several cancer

cell lines. For this, they performed a genome-wide CRISPR/Cas9 study using sensitive cancer cells to identify genes which when deleted, confer resistance to the compound. From both a technical and analytical standpoint, this is simpler than the converse approach of searching for genes which when deleted render resistant cancer cells sensitive to IMT1. In a second approach, they used mithridatism, a procedure based on chronic exposure of sensitive cells to increasing doses of IMT1 such that they develop resistance to the compound. These approaches have identified several interesting candidates, including the VHL-HIF1a axis and mTORC1, which potentially could provide interesting targets for combination therapy with IMTs. In addition to providing targets for cancer therapy, these studies reveal new interactions between mitochondrial transcription and cell metabolism.

Overall the results are sound, the experiments are well controlled and the results interesting. However, there are a few points that should be clarified, as described below. Furthermore, the authors should discuss the possibility that the resistance acquired by cancer cells towards IMT1 may vary according to cancer cell types and that it may be misleading to extrapolate their conclusions to all IMT1-resistant cancer cells.

Specific comments:
1-IMT1 blocks mtDNA transcription, which results in depletion of the respiratory chain complexes. Is this the cause of decreased cell proliferation or could the inhibitors exhibit other, off-target effects? I am not sure that this was established in the initial paper (Bonekamp et al., 2020). The question could be addressed using Rho0 cells. In addition, using chloramphenicol (CAP), the authors show that OXPHOS complexes, at least COX2, are decreased, yet the cells do not die and are able to proliferate normally (suppl. Fig 2). This again raises the question of the mechanisms underlying cell proliferation inhibition and/or apoptosis. Is it due to decreased oxygen consumption, to decreased ATP production, to loss of mtRNAs, or to some other mechanism? Do the authors have an idea what could be the main cause leading to decreased cell proliferation/apoptosis? Finally, it is not clear whether IMT1 triggers apoptosis or decrease cell division or both.

2-Rapamycin partially restores respiratory chain complexes and makes RKO cancer cells resistant to IMT1. Does rapamycin restore oxygen consumption rates or is the resistance mediated by an OCR-independent mechanism as is probably the case for FG4592?

3-Is HIF1a expression increased in IMT1 treated resistant cancer cells?

4-The effect of rapamycin on HeLa cells is minimal (Figure 1H). Amongst the IMT1 sensitive cell lines, it would be useful to know what proportion becomes resistant upon HIF1a activation and mTOR inhibition. In addition to RKO, HeLa and miaPACA-2 cells, it would be interesting if the authors could test a few additional IMT1-sensitive cell lines, providing this could be done within a reasonable time-frame.

5-The authors claim that inhibition of mitochondrial translation could sensitize cancer cells to IMT1. Again, if not too time-consuming, could the authors test a few of the resistant cancer cell lines reported in their previous paper with IMT1+CAP? Furthermore, given the potential interest of IMT1 as a therapeutic, it would be important to demonstrate that the combination of IMT1+a mitochondrial translation inhibitor does not impair growth and survival of primary, non cancer cells.

Minor comments:
1-The authors claim a synergy between CAP and IMT1. I would agree with this for the RKO cells (Figure 3H). However in my opinion the results presented in Suppl. figure 2E do not clearly support this statement. At best, they support an additive effect of the 2 compounds. Furthermore, in Figure 3I, the combination of IMT1 and CAP on IMT1 resistant cells is very modest. I would suggest that the authors be more cautious in their interpretation of these results.

2-Because of the link between HIF1a and glycolysis, it could be useful to test whether reducing glycolysis, with 2-deoxyglucose for example, would sensitize cancer cells to IMT1. Interestingly, 2-DG has been tested in clinical trials for cancer therapy.

Referee #3:

In this study, Mennuni et al. perform a CRISPR-Cas9 based genetic screen to identify genes and pathways which confer resistance to small-molecule inhibitors of mitochondrial transcription (IMTs). Recently developed IMTs represent a novel pharmacological means to manipulate mitochondrial biology in a variety of contexts, such as cancer. Given that, this manuscript will likely be of interest to many across biological disciplines. As presented, the genetic screen performed in this study did not uncover any novel genes or pathways which confer resistance or sensitivity to IMTs. Rather, the work highlighted the previously appreciated roles of MTORC1 and VHL in mitochondrial dysfunction. These findings, nevertheless, are important as these findings may have been expected but never previously demonstrated with this compound. Through the generation of cells resistant to IMTs, the authors also describe an increase in steady-state mtDNA levels as a resistance mechanism to circumvent inhibition of mitochondrial transcription. These findings are more interesting, and this reviewer believes the manuscript in its current form would be elevated by a deeper exploration of the mechanistic underpinnings of these observations.

Major Points:
1. In Figure 1, the authors should demonstrate that FG4592 also rescues expression of mtDNA encoded when cells are treated with IMT1 (as was shown in Figure 1I with rapamycin).
2. In Figure 2F, the authors demonstrate that mtDNA levels are higher in resistant cells than RKO cells when both are treated with IMT1. What about when untreated? The authors should also include this data if they want to make the assertation that steady state levels of mtDNA are higher in resistant cells. Though unlikely, one could argue that they are not in fact higher at steady state levels but instead increase mtDNA replication in response to IMT1 treatment given the data currently presented.
3. Similar to point 2 above, in Figure 2E, the authors should display mtDNA transcript levels in untreated RKO and Resistant cells. It is possible that resistant cells have increased mtDNA levels but no difference in mtDNA transcripts. If this is a case, it suggests that the excess mtDNA only is functionally relevant when IMT1 is used to inhibit mitochondrial transcription.
a. This data would also help interpret the observation that steady state levels of mtDNA encoded proteins in RKO and resistant cells are the same (Figure 2B, DMSO vs. DMSO). This data suggests that perhaps there is no functional effect of increased mtDNA levels in resistant cells when not challenged with inhibition of mitochondrial transcription.
4. The identification of mitochondrial translation as liability of resistant cells is a logical avenue of investigation stemming form the proteomic data and CRISPR screen in RKO cells. The data in 2D and 3D is nice and emphasizes this point. Do the authors believe/have data that this is directly related to the compensatory increase in mtDNA levels seen in resistant cells?
5. This reviewer believes there could be more done to mechanistically understand how the resistant cells are resistant to IMT1. If the authors believe increased mtDNA levels are responsible for resistance, manipulation of mtDNA levels should be able to revert sensitivity of the cells (ie genetic manipulation of POLG). Perhaps beyond the scope of this study, ideally the authors could perform a whole genome CRISPR-screen in resistant cells to identify unique liabilities absent from parental cells.

Minor Points:
1. It is unclear what the controls mentioned in the proteomics data (Figure 3E,F) are they RKO and Resistant cells treated with DMSO? If so, it would be interesting to include this data as well to be able to discern base line differences in the proteome of parental and resistant cells.
2. Without the data requested in Major points 2 and 3, the authors must qualify the following sentence in the discussion (lines 306-308) as they have only shown this in the case of IMT1 inhibition:
"We rather found a compensatory boost of mitochondrial function led by increased mtDNA levels, transcript and protein levels"
3. In the introduction (line 57) the authors state that protein coding genes are mostly spared from mutations in cancer. This reviewer does not fully agree with this statement. Rather, mtDNA seems to be spared from loss of function mutations at high levels of heteroplasmy. mtDNA mutations are in fact common across tumor types (PMID: 33833465, 32024997). The language in the introduction should be modified to reflect this nuance.
4. Using pharmacological approaches, the authors convincingly validate MTORC1 and VHL as positive hits from their genetic screen. Given these pathways were identified by CRISPR-Cas9 screening, it would be nice to additionally validate these findings/hits using genetic approaches with sgRNAs from the screen itself.
5. In Figure 3F, there are several proteins which are differentially changed in RKO and Resistant cells. It would be more interesting for the reader to also label proteins whose relative expression changes are different in the two cell lines.
6. Figure 1A and Figure 2A, the x axis should be labeled as IMT1 concentration.

We thank the reviewers for their constructive feedback and suggestions to improve the manuscript. We have now performed extensive sets of additional experiments to address the concerns of the reviewers.

Referee #1:

The manuscript by Mennuni et al. investigates resistance mechanisms to inhibitors of mitochondrial transcription (IMTs). IMTs were previously discovered by the same group to robustly inhibit POLRMT mtDNA gene expression. In their previous studies, the authors found anti-tumor properties of IMTs and in the present manuscript they employed CRISPR-Cas9 screening to search for pathways involved in resistance to IMTs treatment. Authors found VHL and mTORC1 pathways to be involved in IMTs resistance and they showed that IMTs resistance relies on upregulation of mitochondrial gene expression. Overall I find the paper straightforward to read, I like the focused CRISPR results, and points to resistance mechanisms which are important to be cognizant of in the context of future translational studies.

Comments:
1. Authors found VHL and mTORC1 pathway inhibition as a source of IMTs resistance. What would be a proposed mechanism of the resistance? Since authors observe increased levels of mitochondrial oxphos proteins by rapamycin treatment is it by decreased mitophagy, increased mitochondrial biogenesis or altered protein stability? Are the mechanisms convergent or distinct between vHL and mTOR? Is it clear why vHL was previously identified in CRISPR screens but mTOR pathway was not? I would love to see the authors develop a bit more the mechanisms here linking VHL or mTOR and mitochondria.
We investigated whether increased mitochondrial biogenesis may explain the increased tolerance to IMT1 treatment in presence of rapamycin and FG4592. We found no differences in mtDNA copy number or mtDNA transcript levels in RKO cells treated with IMT1 and rapamycin or FG4592 (new Figure EV3A, B). Moreover, there was no induction of expression of PGC1α which is considered a master regulator of mitochondrial biogenesis (new Figure EV3C). Similarly, rapamycin did not increase mtDNA and mtRNA levels in MiaPaCa-2 and HeLa cells during IMT1 treatment (new Figure EV3D-G). Therefore, our data do not support that increased mitochondrial biogenesis is responsible for the IMT1 resistance conferred by rapamycin or FG4592 treatment.

We assessed the production rate and stability of mtDNA-encoded OXPHOS proteins by performing mitochondrial translation assays in whole cells. We found a mild increase in both the production rate and stability of newly synthesized mitochondrial proteins in cells treated with IMT1 in the presence of rapamycin (new Figure 3A, B). In cells treated with IMT1 in the presence of FG4592, no significant changes were observed (new Figure 3A, B). Consistent with these results, there was an increase of steady-state levels of the mtDNA-encoded COX2 protein and the oxygen consumption rate (OCR) in cells treated with IMT1 and rapamycin, whereas no significant changes were observed after FG4592 treatment (new Figure 3C-G). We thus conclude that rapamycin increases mitochondrial protein synthesis and protein stability and thereby promotes cellular respiration. It should be noted that a mild increase in mitochondrial function can have a great effect on cell survival. It is well known from mitochondrial disorders that OXPHOS capacity slightly below or above a cellular threshold can have dramatic consequences on cellular function and survival (Stewart and Chinnery, 2015).

We estimated the cellular autophagic flux by assessing the accumulation of LC3BII after the inhibition of lysosomal acidification with ammonium chloride and observed a marked decrease in LC3BII levels in presence of IMT1 (new Figure EV3H, I). Autophagy is typically activated by cellular starvation and it is to some extent unexpected that autophagy is reduced in IMT1-treated cells. However, the autophagy process itself is energy dependent and it is possible that the severe cellular energy depletion induced by IMT1 treatment does not allow activation of autophagy to proceed normally. Consistent with this way of reasoning, we found that neither rapamycin nor FG4592 could normalize the reduced autophagic flux caused by IMT1 treatment. We therefore conclude that the protective action of rapamycin and FG4592 is not caused by normalization of autophagy in IMT1-treated cells (new Figure EV3H, I).

Based on the results above, we conclude that rapamycin and FG4592 confers resistance to IMT1 via different mechanisms. Rapamycin stimulates mitochondrial protein synthesis, stabilizes mitochondrial proteins and promotes respiration, whereas FG4592 does not have these effects. FG4592 is known to promote oxygen-independent survival mechanisms by stabilizing HIF1α as we also show here (new Figure 2E), and it is known to decrease cellular reliance on mitochondrial respiration (Papandreou *et al.*, 2006; Zhang *et al.*, 2007).

2. Introduction lines 55-58: In contrast to the author's note, only in certain tumor types (colorectal, renal, thyroid) have mtDNA mutations shown to be enriched with VAF patterns and signatures of selection compatible with "driver" properties (Gammage and Frezza, BMC, 2019, Gorelick et al., Nat Metab., 2021, Gopal et al., PNAS, 2018). Utility of IMTs for cancer treatment may be restricted to this subset.
We agree that the role of mtDNA mutations in tumors is complex and we have included additional references and text to better explain this in the introduction.

3. Results lines 88-90: Authors note that one third of previously studied cancer cell lines are sensitive to IMTs. It would be interesting to add a line of comment why these particular cell lines are sensitive while other are not -- can they now go back and explain this on the basis of either the VHL or MTOR pathway?
We agree with the reviewer that this is an interesting point. In addition to the three cell lines tested in the first version of the manuscript, we have now included four additional IMT1-sensitive cell lines. We thus present results from seven cancer cell lines representing four tissues of origin (colon, lung, uterine cervix and pancreas). We found that rapamycin can counteract IMT1-induced cell death in five of the seven tested cell lines (new Figure 2B-D, I and EV2B-E). FG4592 improved IMT1 tolerance in four of the seven cell lines tested (new Figure 2F-H, J and EV2F-I). These results show that IMT1-resistance mechanisms are conserved in many, but not all cancer cell lines. Given the known genetic heterogeneity of cancers, it is not unexpected that resistance mechanisms may vary in cancers sensitive to a particular treatment like IMT1.

4. Did the authors study if IMT1-resistant cells rely on oxphos vs glycolysis? How does it compare to IMT1-sensitive cells and cells with VHL
We have now performed Seahorse characterization of OXPHOS in RKO cells treated with IMT1 in combination with rapamycin or FG4592. Rapamycin mildly improved the oxygen consumption rate (OCR) and reduced the shift towards glycolytic metabolism in the presence of IMT1 (new Figure 3E, F). In contrast, FG4592 treatment did not impact mitochondrial respiration or alter the glycolytic contribution to cellular metabolism in IMT1-treated cells (new Figure 3E, G).

We also characterized cellular bioenergetics of RKO cells and IMT1-resistant RKO cells. The extracellular acidification rate (ECAR), reflecting glycolysis, in resistant RKO cells was similar to the ECAR in the RKO cells and only showed a minor increase upon IMT1 treatment. In contrast, the ECAR drastically increased when the parental RKO cells were treated with IMT1 (new Figure 5A, B). Moreover, the sensitivity to 2-deoxyglucose, an inhibitor of glycolysis, was similar in RKO cells and IMT1-resistant RKO cells (new Figure 5C). These findings show that increased glycolysis does not explain the resistance phenotype.

The basal OCR was lower in the IMT1-resistant RKO cells in comparison with RKO cells (new Figure 4H). IMT1 treatment led to marked decrease in the basal OCR in RKO cells, whereas the decrease was much smaller IMT1-resistant RKO cells (new Figure 4H).

Referee #2:

The authors have recently developed small molecule inhibitors of the mitochondrial RNA polymerase (POLMRT)(Bonemkamp et al., 2020). These inhibitors of mitochondrial transcription (IMTs) repress cancer cell proliferation in vitro and delay tumour growth in xenografts of human cancer cells in the mouse. However, from a panel of 89 cancer cell lines, they found that approximately two thirds were resistant to IMTs.

In the present paper, the authors study the mechanisms underlying resistance to their lead compound IMT1 in several cancer cell lines. For this, they performed a genome-wide CRISPR/Cas9 study using sensitive cancer cells to identify genes which when deleted, confer resistance to the compound. From both a technical and analytical standpoint, this is simpler than the converse approach of searching for genes which when deleted render resistant cancer cells sensitive to IMT1. In a second approach, they used mithridatism, a procedure based on chronic exposure of sensitive cells to increasing doses of IMT1 such that they develop resistance to the compound. These approaches have identified several interesting candidates, including the VHL-HIF1a axis and mTORC1, which potentially could provide interesting targets for combination therapy with IMTs. In addition to providing targets for cancer therapy, these studies reveal new interactions between mitochondrial transcription and cell metabolism.

Overall the results are sound, the experiments are well controlled and the results interesting. However, there are a few points that should be clarified, as described below. Furthermore, the authors should discuss the possibility that the resistance acquired by cancer cells towards IMT1 may vary according to cancer cell types and that it may be misleading to extrapolate their conclusions to all IMT1-resistant cancer cells.
We agree that it is unlikely that resistance mechanisms to IMT1 will be universally conserved in cancer cell lines and we have now tested additional cell lines. For details, see comment to reviewer 1, point 3.

Specific comments:
1-IMT1 blocks mtDNA transcription, which results in depletion of the respiratory chain complexes. Is this the cause of decreased cell proliferation or could the inhibitors exhibit other, off-target effects? I am not sure that this was established in the initial paper (Bonekamp et al., 2020). The question could be addressed using Rho0 cells.
We have extensively characterized the mode of action of IMTs in our previous paper by Bonekamp et al., *Nature* 2020. IMT1 selectively binds a pocket in the mitochondrial RNA polymerase (POLRMT) and acts as a site-specific allosteric regulator of enzyme activity, as demonstrated by a cryo-EM structure of POLRMT with IMT1 bound (Fig.2d-f in Bonekamp et al.). We also performed a random mutagenesis experiment to select cells resistant to IMT treatment and found that POLRMT is the only in vivo target (Fig.2a-c in Bonekamp et al). Based on these random mutagenesis results, we also used CRISPR-Cas9 to introduce the resistance mutations into the *POLRMT* gene and showed that such cells became resistant to IMT treatment (Fig. 2a-c, Bonekamp et al.). Furthermore, we performed temporal proteomics experiments of IMT-treated cell lines and found that the pattern of altered protein expression was fully consistent with IMT selectively inhibiting POLRMT (Extended data Fig. 2d, Bonekamp et al.). Finally, we also tested a range of other polymerases (yeast RPO41, T7 phage RNA polymerase, *E. coli* RNA polymerase, mammalian nuclear RNA polymerase I, II and III, and mammalian mitochondrial DNA polymerase) and found that IMT treatment had no effect on their enzymatic activities (Extended data Fig. 3a-e, Bonekamp et al.). On the basis of these results, we feel confident that IMTs are highly specific inhibitors of POLRMT that do not cause main off-target effects.

In addition, using chloramphenicol (CAP), the authors show that OXPHOS complexes, at least COX2, are decreased, yet the cells do not die and are able to proliferate normally (suppl. Fig 2).
We carefully titrated CAP concentration and showed that high CAP doses indeed induce cell death (new Figure EV4A, E). To allow studies of the synergy between IMT1 and CAP treatment, we intentionally chose a CAP concentration that impaired mitochondrial translation, without inducing cell death.

It is also important to recognize that CAP acts at the level of the ribosome, whereas the IMTs act further upstream, at the level of mtDNA transcription. Although the levels of COX2 protein are decreased to similar levels in CAP and IMT1-treated cells, there are additional defects in the IMT1-treated cells, such as low tRNA levels, low rRNA levels (affecting mitoribosomal biogenesis) and low mRNA levels, which further decrease the robustness of the mtDNA expression system.

This again raises the question of the mechanisms underlying cell proliferation inhibition and/or apoptosis. Is it due to decreased oxygen consumption, to decreased ATP production, to loss of mtRNAs, or to some other mechanism? Do the authors have an idea what could be the main cause leading to decreased cell proliferation/apoptosis? Finally, it is not clear whether IMT1 triggers apoptosis or decrease cell division or both.
In our previous paper, we reported that IMT treatment induces a bioenergetic crisis, which leads to severe depletion of key cellular metabolites, reduced cellular proliferation and eventually apoptotic cell death (see Fig. 3c-g and Extended Fig. 7 in Bonekamp et al. 2020). We have now performed metabolomics in parental and IMT1-resistant RKO cells treated with IMT1 for 96 hours (new Figure 6C and Dataset EV3). The IMT1 treated cells developed depletion of a range of metabolites in a pattern consistent with our previous description of metabolite changes in IMT1-treated A2780 ovarian cancer cells (Bonekamp et al., 2020). Interestingly, the IMT1-resistant RKO cell line maintained these metabolites at higher levels after IMT1 treatment (new Figure 6C and Dataset EV3), showing that the ability to avoid a cellular energy crisis is an important mechanism for survival.

2-Rapamycin partially restores respiratory chain complexes and makes RKO cancer cells resistant to IMT1. Does rapamycin restore oxygen consumption rates or is the resistance mediated by an OCR-independent mechanism as is probably the case for FG4592?
We found that respiration rates were partially restored by rapamycin, but not by FG4592, in IMT1-treated RKO cells. For additional details, see also response to Referee #1, point 1 and 4.

3-Is HIF1a expression increased in IMT1 treated resistant cancer cells?
We have assessed the changes in protein expression in RKO cells and IMT1-resistant RKO cells by proteomics and did not detect the HIF1α protein (Dataset EV1). Our data argue that the IMT1-induced resistance is explained by increases of mtDNA copy number, mitochondrial transcripts, mitochondrial protein synthesis of mtDNA-encoded OXPHOS proteins. These changes lead to improved levels of cellular metabolites (new Figures 4A-H and 6C). We thus conclude that increased mtDNA expression (and not activation of hypoxic responses) explains why the IMT1-resistant RKO cells tolerate IMT1 treatment.

4-The effect of rapamycin on HeLa cells is minimal (Figure 1H). Amongst the IMT1 sensitive cell lines, it would be useful to know what proportion becomes resistant upon HIF1a activation and mTOR inhibition. In addition to RKO, HeLa and miaPACA-2 cells, it would be interesting if the authors could test a few additional IMT1-sensitive cell lines, providing this could be done within a reasonable time-frame.
In addition to the three previously tested cell lines, we now included four additional IMT1-sensitive cell lines. For details, see response to Referee #1, point 3.

5-The authors claim that inhibition of mitochondrial translation could sensitize cancer cells to IMT1. Again, if not too time-consuming, could the authors test a few of the resistant cancer cell lines reported in their previous paper with IMT1+CAP?
As suggested, we have included additional cell lines to investigate whether CAP treatment may sensitize cells that are insensitive to IMT treatment. We found that treatment with 1□g/ml CAP treatment sensitized cells to IMT1 to different extents. A combination of IMT1 and CAP treatment had a strong impact on IMT1-resistant RKO cells, but milder effects on Panc1, Capan2 and HCT-29 cells, and did not affect Calu6 cells (new Figure 7D). A higher CAP concentration (100□g/ml) resulted in increased sensitivity to IMT1 (new Figure EV4G). It should be noted that a high CAP concentration can induce cell death on its own, whereas this was not the case at a lower CAP concentration (new Figure EV4A, E). These data show that simultaneous targeting of mitochondrial transcription and translation have a synergistic effect in some cancer cells, whereas others are unaffected. We point this out in the discussion of the manuscript.

Furthermore, given the potential interest of IMT1 as a therapeutic, it would be important to demonstrate that the combination of IMT1+a mitochondrial translation inhibitor does not impair growth and survival of primary, non cancer cells.
We used a combination of IMT1 and CAP to treat primary human fibroblasts from two healthy donors and found no effect on cell viability (new Figure EV4D).

Minor comments:

1-The authors claim a synergy between CAP and IMT1. I would agree with this for the RKO cells (Figure 3H). However in my opinion the results presented in Suppl. figure 2E do not clearly support this statement. At best, they support an additive effect of the 2 compounds. Furthermore, in Figure 3I, the combination of IMT1 and CAP on IMT1 resistant cells is very modest. I would suggest that the authors be more cautious in their interpretation of these results.
We agree with the reviewer and now use a more cautious wording.

2-Because of the link between HIF1a and glycolysis, it could be useful to test whether reducing glycolysis, with 2-deoxyglucose for example, would sensitize cancer cells to IMT1. Interestingly, 2-DG has been tested in clinical trials for cancer therapy.
We found that the sensitivity to 2-deoxyglucose was similar in RKO cells and IMT1-resistant RKO cells (new Figure 5C). Furthermore, IMT1 treatment produced a smaller shift towards glycolytic metabolism in the IMT1-resistant RKO cells in comparison with RKO cells (new Figure 5B). Based on these data, we conclude that increased reliance on glycolysis is not a likely mechanism of resistance in the IMT1- resistant RKO cells.

Referee #3:

In this study, Mennuni et al. perform a CRISPR-Cas9 based genetic screen to identify genes and pathways which confer resistance to small-molecule inhibitors of mitochondrial transcription (IMTs). Recently developed IMTs represent a novel pharmacological means to manipulate mitochondrial biology in a variety of contexts, such as cancer. Given that, this manuscript will likely be of interest to many across biological disciplines. As presented, the genetic screen performed in this study did not uncover any novel genes or pathways which confer resistance or sensitivity to IMTs. Rather, the work highlighted the previously appreciated roles of MTORC1 and VHL in mitochondrial dysfunction. These findings, nevertheless, are important as these findings may have been expected but never previously demonstrated with this compound. Through the generation of cells resistant to IMTs, the authors also describe an increase in steady-state mtDNA levels as a resistance mechanism to circumvent inhibition of mitochondrial transcription. These findings are more interesting, and this reviewer believes the manuscript in its current form would be elevated by a deeper exploration of the mechanistic underpinnings of these observations.

Major Points:
1. In Figure 1, the authors should demonstrate that FG4592 also rescues expression of mtDNA encoded when cells are treated with IMT1 (as was shown in Figure 1I with rapamycin).
We have now performed western blot analyses of COX2 levels in the presence of rapamycin or FG4592 (new Figure 3C, D). We show that rapamycin partly rescues the mt-COX2 steady state levels, whereas FG4592 has no such effect. See also response to Reviewer #1, point 1.

2. In Figure 2F, the authors demonstrate that mtDNA levels are higher in resistant cells than RKO cells when both are treated with IMT1. What about when untreated? The authors should also include this data if they want to make the assertation that steady state levels of mtDNA are higher in resistant cells. Though unlikely, one could argue that they are not in fact higher at steady state levels but instead increase mtDNA replication in response to IMT1 treatment given the data currently presented.
We apologize for not presenting our data in a clearer fashion and we have now revised the presentation of data (new Figure 4G). The figure shows that the mtDNA levels are not higher in the IMT1-resistant RKO cells in comparison with the parental RKO cells. However, when IMT1

treatment is administered the mtDNA levels drop markedly in the parental RKO cells, whereas a lesser decrease is observed in the IMT1-resistant RKO cells (new Figure 4G).

3. Similar to point 2 above, in Figure 2E, the authors should display mtDNA transcript levels in untreated RKO and resistant cells. It is possible that resistant cells have increased mtDNA levels but no difference in mtDNA transcripts. If this is a case, it suggests that the excess mtDNA only is functionally relevant when IMT1 is used to inhibit mitochondrial transcription.
We have now updated the figure as requested (new Figure 4F). The new figure shows that the transcript levels are not higher in the IMT1-resistant RKO cells in comparison with RKO cells. However, when IMT1 treatment is administered, the transcript levels drop markedly in RKO cells, whereas no decrease is observed in the IMT1-resistant RKO cells (new Figure 4E). IMT1 treatment of IMT1-resistant RKO cells thus results in moderate decrease of mtDNA and no decrease in mitochondrial transcripts, whereas RKO cells show a profound drop in both mtDNA and mitochondrial transcripts.

a. This data would also help interpret the observation that steady state levels of mtDNA encoded proteins in RKO and resistant cells are the same (Figure 2B, DMSO vs. DMSO). This data suggests that perhaps there is no functional effect of increased mtDNA levels in resistant cells when not challenged with inhibition of mitochondrial transcription.
As there is no difference in levels of mtDNA and mitochondrial transcripts in RKO cells and IMT1-resistant RKO cells treated with DMSO (point 2 and 3 above), no difference in protein abundance is expected. The difference between the two cell lines occurs only in presence of IMT1, which causes a strong reduction in mitochondrial proteins in RKO cells, but not in the IMT1-resistant RKO cells.

4. The identification of mitochondrial translation as liability of resistant cells is a logical avenue of investigation stemming form the proteomic data and CRISPR screen in RKO cells. The data in 2D and 3D is nice and emphasizes this point. Do the authors believe/have data that this is directly related to the compensatory increase in mtDNA levels seen in resistant cells? This reviewer believes there could be more done to mechanistically understand how the resistant cells are resistant to IMT1. If the authors believe increased mtDNA levels are responsible for resistance, manipulation of mtDNA levels should be able to revert sensitivity of the cells (ie genetic manipulation of POLG). Perhaps beyond the scope of this study, ideally the authors could perform a whole genome CRISPR-screen in resistant cells to identify unique liabilities absent from parental cells.
The levels of mitochondrial transcription factor A (TFAM) directly controls the levels of mtDNA and we therefore decreased TFAM expression by using two different siRNAs. Knockdown of TFAM in resistant RKO cells causes a gradual decrease of mtDNA copy number (new Figure EV4I, J). The siRNA that had the most profound effect on mtDNA copy number also decreased cell viability of IMT1-treated IMT1- resistant RKO cells (new Figure 7E-G). We thus conclude that reduced mtDNA copy number increases the sensitivity to IMT1 treatment in IMT1-resistant RKO cells.

Minor Points:

1. It is unclear what the controls mentioned in the proteomics data (Figure 3E,F) are they RKO and Resistant cells treated with DMSO? If so, it would be interesting to include this data as well to be able to discern base line differences in the proteome of parental and resistant cells.
The RKO cells underwent long-term treatment with DMSO (to generate controls) or with IMT1 (to generate IMT1-resistant cells). This explains why there is a single control (DMSO-treated RKO cells) that is relevant for comparison with IMT1-treated non-resistant and resistant RKO cells. We used the same type of controls for the new metabolomics data that we present in the revised manuscript. See also major point 2 and 3 above.

2. Without the data requested in Major points 2 and 3, the authors must qualify the following sentence in the discussion (lines 306-308) as they have only shown this in the case of IMT1 inhibition:
"We rather found a compensatory boost of mitochondrial function led by increased mtDNA levels, transcript and protein levels"

We have altered the wording as suggested. See also major point 2 and 3 above.

3. In the introduction (line 57) the authors state that protein coding genes are mostly spared from mutations in cancer. This reviewer does not fully agree with this statement. Rather, mtDNA seems to be spared from loss of function mutations at high levels of heteroplasmy. mtDNA mutations are in fact common across tumor types (PMID: 33833465, 32024997). The language in the introduction should be modified to reflect this nuance.
We agree with these comments and have changed the introduction to make these points clearer.

4. Using pharmacological approaches, the authors convincingly validate MTORC1 and VHL as positive hits from their genetic screen. Given these pathways were identified by CRISPR-Cas9 screening, it would be nice to additionally validate these findings/hits using genetic approaches with sgRNAs from the screen itself.
We feel that the screen itself represents a comprehensive and unbiased genetic tool to identify genes and pathways of interest. It should be emphasized that we found multiple hits in both of these pathways when performing the CRISPR-Cas9 screen (new Figure 1F and Dataset EV2). Also, as the reviewer points out, the pharmacological interventions with rapamycin and FG4592 validates the identified hits.

5. In Figure 3F, there are several proteins which are differentially changed in RKO and Resistant cells. It would be more interesting for the reader to also label proteins whose relative expression changes are different in the two cell lines.
We have updated the graph as suggested (new Figure 6B, dataset EV1). A full list of differentially represented proteins is also available as raw data in the supplementary material and has been deposited in the ProteomeXchange Consortium via PRIDE partner repository database (see data availability section below).

6. Figure 1A and Figure 2A, the x axis should be labeled as IMT1 concentration.
We have updated the figures as suggested. The previous Figure 2A is Figure 4A in the revised manuscript.

Data Availability
The mass spectrometry proteomics data have been deposited to the ProteomeXchange Consortium via PRIDE partner repository with the dataset identifier PXD026481. Please find the credential for access the database below:

REDACTED

1st Revision - Editorial Decision 8th Oct 2021

Dear Nils,

Thank you for submitting your revised manuscript. It has now been seen by two of the original referees.

As you can see, the referees find that the study is significantly improved during revision and recommends publication. However, I need you to address the editorial points below before I can accept the manuscript.

• Please make the following dataset publicly available: https://www.ebi.ac.uk/pride/archive/projects/PXD026481
• The figure legends should be moved to after the Reference list. The tables should be at the end of the file.
• We note that DATASET EV legends currently do not include the file names - e.g. Dataset EV1, next to the title in the legend.
• Papers published in EMBO Reports include a 'synopsis' and 'bullet points' to further enhance discoverability. Both are displayed on the html version of the paper and are freely accessible to all readers. The synopsis includes a short standfirst summarizing the study in 1 or 2 sentences that summarize the paper (max 35 words) and are provided by the authors and streamlined by the handling editor. I would therefore ask you to include your synopsis blurb and 3-5 bullet points listing the key experimental findings.
• Our production/data editors have asked you to clarify several points in the figure legends (see attached document). Please incorporate these changes in the attached word document and return it with track changes activated.

Thank you again for giving us to consider your manuscript for EMBO Reports, I look forward to your minor revision.

Kind regards,

Deniz

--
Deniz Senyilmaz Tiebe, PhD
Editor
EMBO Reports

Referee #2:

The authors have answered my previous questions and their paper has been significantly strengthened.

Referee #3:

In this revised manuscript, Mennuni et al. appropriately addressed the concerns of this reviewer and the two additional reviewers. The manuscript has been significantly improved during the revision process and is suitable for publication.

As I mentioned in my first comments to the authors, I would have liked to see the authors validate the results of their CRISPR screen using genetics in addition to the chemical approaches used here. I understand their assertation that a CRISPR genetic screen is a comprehensive tool to identify relevant targets and the validation using chemical approaches is sufficient.

That being said, I firmly believe it is best practice to validate the top hits of a CRISPR screen using the sgRNA from the screen regardless of other methods used to substantiate the findings. Simply validating the single top scoring genee from the respective mTOR and VHL pathways would suffice. The ease by which the authors could have done these validation experiments but have not is concerning. It leads me to speculate that the authors may have tried to validate their screen results with single genetic perturbations but were unsuccessful.

Nevertheless, I support the publication of this manuscript as is.

The authors have addressed all minor editorial requests.

2nd Revision - Editorial Decision 19th Oct 2021

Dear Nils,

Thank you for submitting your revised manuscript. I have now looked at everything and all is fine. Therefore, I am very pleased to accept your manuscript for publication in EMBO Reports.

Congratulations on a nice work!

Kind regards,

Deniz
--
Deniz Senyilmaz Tiebe, PhD
Editor
EMBO Reports

--

At the end of this email I include important information about how to proceed. Please ensure that you take the time to read the information and complete and return the necessary forms to allow us to publish your manuscript as quickly as possible.

As part of the EMBO publication's Transparent Editorial Process, EMBO reports publishes online a Review Process File to accompany accepted manuscripts. As you are aware, this File will be published in conjunction with your paper and will include the referee reports, your point-by-point response and all pertinent correspondence relating to the manuscript.

If you do NOT want this File to be published, please inform the editorial office within 2 days, if you have not done so already, otherwise the File will be published by default [contact: emboreports@embo.org]. If you do opt out, the Review Process File link will point to the following statement: "No Review Process File is available with this article, as the authors have chosen not to make the review process public in this case." Please note that the author checklist will still be published even if you opt out of the transparent process.

Thank you again for your contribution to EMBO reports and congratulations on a successful publication. Please consider us again in the future for your most exciting work.

****

THINGS TO DO NOW:

You will receive proofs by e-mail approximately 2-3 weeks after all relevant files have been sent to our Production Office; you should return your corrections within 2 days of receiving the proofs.

Please inform us if there is likely to be any difficulty in reaching you at the above address at that time. Failure to meet our deadlines may result in a delay of publication, or publication without your corrections.

All further communications concerning your paper should quote reference number EMBOR-2021-53054V3 and be addressed to emboreports@wiley.com.

Should you be planning a Press Release on your article, please get in contact with emboreports@wiley.com as early as possible, in order to coordinate publication and release dates.

EMBO PRESS

YOU MUST COMPLETE ALL CELLS WITH A PINK BACKGROUND ↓

PLEASE NOTE THAT THIS CHECKLIST WILL BE PUBLISHED ALONGSIDE YOUR PAPER

Corresponding Author Name: Nils-Göran Larsson
Journal Submitted to: EMBO Reports
Manuscript Number: EMBOR-2021-53054V1

Reporting Checklist For Life Sciences Articles (Rev. June 2017)

This checklist is used to ensure good reporting standards and to improve the reproducibility of published results. These guidelines are consistent with the Principles and Guidelines for Reporting Preclinical Research issued by the NIH in 2014. Please follow the journal's authorship guidelines in preparing your manuscript.

A- Figures
1. Data
The data shown in figures should satisfy the following conditions:

- the data were obtained and processed according to the field's best practice and are presented to reflect the results of the experiments in an accurate and unbiased manner.
- figure panels include only data points, measurements or observations that can be compared to each other in a scientifically meaningful way.
- graphs include clearly labeled error bars for independent experiments and sample sizes. Unless justified, error bars should not be shown for technical replicates.
- if n< 5, the individual data points from each experiment should be plotted and any statistical test employed should be justified
- Source Data should be included to report the data underlying graphs. Please follow the guidelines set out in the author ship guidelines on Data Presentation.

2. Captions

Each figure caption should contain the following information, for each panel where they are relevant:

- a specification of the experimental system investigated (eg cell line, species name).
- the assay(s) and method(s) used to carry out the reported observations and measurements
- an explicit mention of the biological and chemical entity(ies) that are being measured.
- an explicit mention of the biological and chemical entity(ies) that are altered/varied/perturbed in a controlled manner.

- the exact sample size (n) for each experimental group/condition, given as a number, not a range;
- a description of the sample collection allowing the reader to understand whether the samples represent technical or biological replicates (including how many animals, litters, cultures, etc.).
- a statement of how many times the experiment shown was independently replicated in the laboratory.
- definitions of statistical methods and measures:
  - common tests, such as t-test (please specify whether paired vs. unpaired), simple χ2 tests, Wilcoxon and Mann-Whitney tests, can be unambiguously identified by name only, but more complex techniques should be described in the methods section;
  - are tests one-sided or two-sided?
  - are there adjustments for multiple comparisons?
  - exact statistical test results, e.g., P values = x but not P values < x;
  - definition of 'center values' as median or average;
  - definition of error bars as s.d. or s.e.m.

Any descriptions too long for the figure legend should be included in the methods section and/or with the source data.

In the pink boxes below, please ensure that the answers to the following questions are reported in the manuscript itself. Every question should be answered. If the question is not relevant to your research, please write NA (non applicable). We encourage you to include a specific subsection in the methods section for statistics, reagents, animal models and human subjects.

USEFUL LINKS FOR COMPLETING THIS FORM

http://www.antibodypedia.com
http://1degreebio.org
http://www.equator-network.org/reporting-guidelines/improving-bioscience-research-repor
http://grants.nih.gov/grants/olaw/olaw.htm
http://www.mrc.ac.uk/Ourresearch/Ethicsresearchguidance/Useofanimals/index.htm
http://ClinicalTrials.gov
http://www.consort-statement.org
http://www.consort-statement.org/checklists/view/32-consort/66-title
http://www.equator-network.org/reporting-guidelines/reporting-recommendations-for-tumo
http://datadryad.org
http://figshare.com
http://www.ncbi.nlm.nih.gov/gap
http://www.ebi.ac.uk/ega
http://biomodels.net/
http://biomodels.net/miriam/
http://jjj.biochem.sun.ac.za
https://osp.od.nih.gov/biosafety-biosecurity-and-emerging-biotechnology/
http://www.selectagents.gov/

B- Statistics and general methods

Please fill out these boxes ↓ (Do not worry if you cannot see all your text once you press return)

1.a. How was the sample size chosen to ensure adequate power to detect a pre-specified effect size?	We aimed to have at least 3 to 5 independent biological replicates for each experiment. The number of idependent experiments is specified in each figure legend.
1.b. For animal studies, include a statement about sample size estimate even if no statistical methods were used.	NA
2. Describe inclusion/exclusion criteria if samples or animals were excluded from the analysis. Were the criteria pre-established?	NA
3. Were any steps taken to minimize the effects of subjective bias when allocating animals/samples to treatment (e.g. randomization procedure)? If yes, please describe.	NA
For animal studies, include a statement about randomization even if no randomization was used.	NA
4.a. Were any steps taken to minimize the effects of subjective bias during group allocation or/and when assessing results (e.g. blinding of the investigator)? If yes please describe.	NA
4.b. For animal studies, include a statement about blinding even if no blinding was done	NA
5. For every figure, are statistical tests justified as appropriate?	Yes
Do the data meet the assumptions of the tests (e.g., normal distribution)? Describe any methods used to assess it.	Normal distribution was analysed in Prism 9 prior to statistical analysis.
Is there an estimate of variation within each group of data?	Data were analysed using Prism 9 and for each statistical analysis the variance for each group was calculated

Is the variance similar between the groups that are being statistically compared?	Yes, in most cases

C- Reagents

6. To show that antibodies were profiled for use in the system under study (assay and species), provide a citation, catalog number and/or clone number, supplementary information or reference to an antibody validation profile. e.g., Antibodypedia (see link list at top right), 1DegreeBio (see link list at top right).	Manufacturer's specifications and catalogue numbers are reported in the Methods section
7. Identify the source of cell lines and report if they were recently authenticated (e.g., by STR profiling) and tested for mycoplasma contamination.	Suppliers (ATCC or DSMZ) and catalogue numbers for all cancer cell lines used are reported in the Methods section. Cell lines were not recently autenticated. Primary human fibroblasts were obtained from the Center of Inherited Metabolic Diseases (CMMS) at the Karolinska University Hospital in conformity with the Regional Ethics Commitee regulations. Cells are routinely tested for mycoplasma in the lab, every four months.

* for all hyperlinks, please see the table at the top right of the document

D- Animal Models

8. Report species, strain, gender, age of animals and genetic modification status where applicable. Please detail housing and husbandry conditions and the source of animals.	NA
9. For experiments involving live vertebrates, include a statement of compliance with ethical regulations and identify the committee(s) approving the experiments.	NA
10. We recommend consulting the ARRIVE guidelines (see link list at top right) (PLoS Biol. 8(6), e1000412, 2010) to ensure that other relevant aspects of animal studies are adequately reported. See author guidelines, under 'Reporting Guidelines'. See also: NIH (see link list at top right) and MRC (see link list at top right) recommendations. Please confirm compliance.	NA

E- Human Subjects

11. Identify the committee(s) approving the study protocol.	NA
12. Include a statement confirming that informed consent was obtained from all subjects and that the experiments conformed to the principles set out in the WMA Declaration of Helsinki and the Department of Health and Human Services Belmont Report.	NA
13. For publication of patient photos, include a statement confirming that consent to publish was obtained.	NA
14. Report any restrictions on the availability (and/or on the use) of human data or samples.	NA
15. Report the clinical trial registration number (at ClinicalTrials.gov or equivalent), where applicable.	NA
16. For phase II and III randomized controlled trials, please refer to the CONSORT flow diagram (see link list at top right) and submit the CONSORT checklist (see link list at top right) with your submission. See author guidelines, under 'Reporting Guidelines'. Please confirm you have submitted this list.	NA
17. For tumor marker prognostic studies, we recommend that you follow the REMARK reporting guidelines (see link list at top right). See author guidelines, under 'Reporting Guidelines'. Please confirm you have followed these guidelines.	NA

F- Data Accessibility

18: Provide a "Data Availability" section at the end of the Materials & Methods, listing the accession codes for data generated in this study and deposited in a public database (e.g. RNA-Seq data: Gene Expression Omnibus GSE39462, Proteomics data: PRIDE PXD000208 etc.) Please refer to our author guidelines for 'Data Deposition'.<br><br>Data deposition in a public repository is mandatory for:<br>a. Protein, DNA and RNA sequences<br>b. Macromolecular structures<br>c. Crystallographic data for small molecules<br>d. Functional genomics data<br>e. Proteomics and molecular interactions	The proteomics data generated in this study have been deposited in PRIDE database and will be available upon acceptance of the manuscript.
19. Deposition is strongly recommended for any datasets that are central and integral to the study; please consider the journal's data policy. If no structured public repository exists for a given data type, we encourage the provision of datasets in the manuscript as a Supplementary Document (see author guidelines under 'Expanded View' or in unstructured repositories such as Dryad (see link list at top right) or Figshare (see link list at top right).	Proteomics data have been deposited in PRIDE, and they are also available as Extended dataset 1. CRISPR-Cas 9 screen results are available as Extended dataset 2 and cellular metabolites changes have been submitted as Extended data 3.
20. Access to human clinical and genomic datasets should be provided with as few restrictions as possible while respecting ethical obligations to the patients and relevant medical and legal issues. If practically possible and compatible with the individual consent agreement used in the study, such data should be deposited in one of the major public access-controlled repositories such as dbGAP (see link list at top right) or EGA (see link list at top right).	NA
21. Computational models that are central and integral to a study should be shared without restrictions and provided in a machine-readable form. The relevant accession numbers or links should be provided. When possible, standardized format (SBML, CellML) should be used instead of scripts (e.g. MATLAB). Authors are strongly encouraged to follow the MIRIAM guidelines (see link list at top right) and deposit their model in a public database such as Biomodels (see link list at top right) or JWS Online (see link list at top right). If computer source code is provided with the paper, it should be deposited in a public repository or included in supplementary information.	NA

G- Dual use research of concern

22. Could your study fall under dual use research restrictions? Please check biosecurity documents (see link list at top right) and list of select agents and toxins (APHIS/CDC) (see link list at top right). According to our biosecurity guidelines, provide a statement only if it could.	NA
